# Comprehensive Chemical Profiling and Mechanistic Insight into Anticancer Activity of *Annona muricata* Leaves Extract

**DOI:** 10.3390/ph17050614

**Published:** 2024-05-10

**Authors:** Rehab H. Abdallah, Al-sayed R. Al-Attar, Youssef M. Shehata, Doaa M. Abdel-Fattah, Rahnaa M. Atta, Omer I. Fantoukh, Ahmed M. Mustafa

**Affiliations:** 1 Department of Pharmacognosy, Faculty of Pharmacy, Zagazig University, Zagazig 44519, Egypt; 2 Department of Pathology, Faculty of Veterinary Medicine, Zagazig University, Zagazig 44519, Egypt; sayedattar50@gmail.com; 3 Department of Biochemistry, Faculty of Veterinary Medicine, Zagazig University, Zagazig 7120001, Egypt; rehabayman4117@gmail.com (Y.M.S.); rahnaaatta@yahoo.com (R.M.A.); 4 Department of Pharmacognosy, College of Pharmacy, King Saud University, P.O. Box 2457, Riyadh 11451, Saudi Arabia; ofantoukh@ksu.edu.sa; 5 Chemistry Interdisciplinary Project (CHIP), School of Pharmacy, University of Camerino, Via Madonna delle Carceri, 62032 Camerino, Italy; ahmed.mustafa@unicam.it

**Keywords:** *Annona muricata*, leaves, UPLC-ESI-MS/MS, immunohistochemistry, molecular biology

## Abstract

The aqueous extract of *Annona muricata* L. leaves was thoroughly analyzed using the UPLC-MS/MS, in addition to a new approach of examination of the extract’s impact on cancer of EAC(Ehrlich ascites carcinoma) in albino male mice. The aim was to investigate the diversity of the phytochemical constituents of the aqueous leaf capsule extract and their impacts on EAC as anticancer agents. The UPLC-ESI-MS/MS screening resulted in 410 tentatively identified metabolites. Among them, 384 compounds were tentatively identified in a previous study, besides a number of 26 compounds belonging to acetogenins, phenolics, flavonoids, alkaloids, and other miscellaneous compounds, which were exclusively identified in the aqueous extract of the leaf capsule. Interestingly, a new compound was tentatively characterized as galloyl-quinic acid-rutinoside. This study also demonstrated that treating EAC mice with an extract from *A. muricata* leaves significantly improved the abnormalities in the expression of pro-apoptotic (Bax and caspase-3) and anti-apoptotic (Bcl-2) genes. Furthermore, the extract showed good protection against induced Ehrlich hepatocarcinoma, according to the microscopical, histological, and immune-histochemical analyses of the liver tissues and tumor mass.

## 1. Introduction

Most plants naturally produce a large variety of bioactive compounds, which may be extracted from various plant parts and have a range of biological activities [1]. Assessing the biological and nutritional aspects of food matrices requires thoroughly identifying the various types of compounds present [2]. The diversity of bioactive substances found in plants is primarily responsible for the differences in active principles found in different plants and even within the same different plant parts from the same species.

*Annona muricata* L., a little evergreen tree that grows to a height of 4 to 8 m and bears green, pointed, heart-shaped fruit, is found in Southeast Asia, Africa, and the tropical regions of South and Central America. It is also known by the names graviola, soursop, and guanabana. Traditionally, a wide range of ailments have been treated with the bark, fruit, seed, and leaves. *A. muricata* leaves can be taken wholly as a nutritional supplement in capsule form or used to make tea, which may have some health benefits [3]. Furthermore, the decoction of the leaf is claimed to have anti-rheumatic and neuralgic properties when administered internally, while the boiled leaves are applied topically to treat rheumatism and abscesses [4]. The leaves of the plant contain significant levels of secondary metabolites, such as glycosaponin, phenolics, and flavonoids, which have been shown to have therapeutic benefits as antioxidant and anticancer potentials [5].

Phytochemical evaluations on different parts of the *A. muricata* plant have shown the presence of various secondary metabolites, including alkaloids, megastigmanes, flavonoltriglycosides, phenolics, cyclopeptides, and essential oils. However, *Annona* species, including *A. muricata*, have been shown to be generally rich sources of annonaceous acetogenin compounds [4,5].

Currently, *A. muricata* leaves are widely utilized by people diagnosed with cancer. According to reports, *A. muricata* is a rich source of acetogenins, which have been shown to possess demonstrated cytotoxic action against various cancer cell types and to be enhanced by the presence of flavonoids to allow for the greatest possible therapeutic effects [4,5].

The ethanolic extract of the whole fruit and aqueous extract of the edible part of the fruit of *A. muricata* were previously investigated and the study showed varying chemical compositions and varying anticancer effects. The difference in the bioactive phytochemicals found in these extracts was mentioned to be the cause of this variation [6].

Despite these studies, *A. muricata* leaves have not experienced detailed phytochemical inspection, especially for their phenolic and flavonoid contents, where most of the studies focused on acetogenins and alkaloids, and our knowledge is comparatively insufficient concerning their potential role in nature. Hence, the attainment of a reasonable perception of natural products necessitates comprehensive investigations of the biological activities of *A. muricata* leaves and their key phytochemicals.

Using gene expression of anti-apoptotic genes, such as Bcl-2 and pro-apoptotic genes such as Bax and caspase-3 in the tumor mass, thorough HPLC–ESI-MS/MS analyses were performed using both negative and positive electrospray ionization modes in this context to analyze the chemical components of the water leaf capsules’ extract and their cytotoxic actions against induced Ehrlich ascites carcinoma in albino adult mice. Moreover, liver tissues and the tumor mass were examined under a microscope by a pathologist and by immunohistochemistry. Our current research is considered the first extensive study of almost all the chemical constituents of the aqueous leaf capsule extract, especially the phenolic constituents that led to the tentative identification of 410 constituents, including a new compound. It is also the first time the aqueous leaf capsule extract underwent immunohistopathology and molecular biology studies. This study revealed that induction with EAC cells resulted in abnormalities in the expression of pro-apoptotic (Bax and caspase-3) and anti-apoptotic (Bcl-2)genes. Moreover, the pathology and immunohistochemistry of the tumor mass and liver tissues showed extensive growth of malignant Ehrlich carcinoma cells and marked degeneration of hepatocytes and infiltration by tumor cells to liver tissue. These abnormalities were markedly ameliorated after treatment in the EAC mice with the *A. muricata* L. aqueous leaf capsule extract.

## 2. Results and Discussion

### 2.1. Phytochemical Characterization

**Figure 1:**
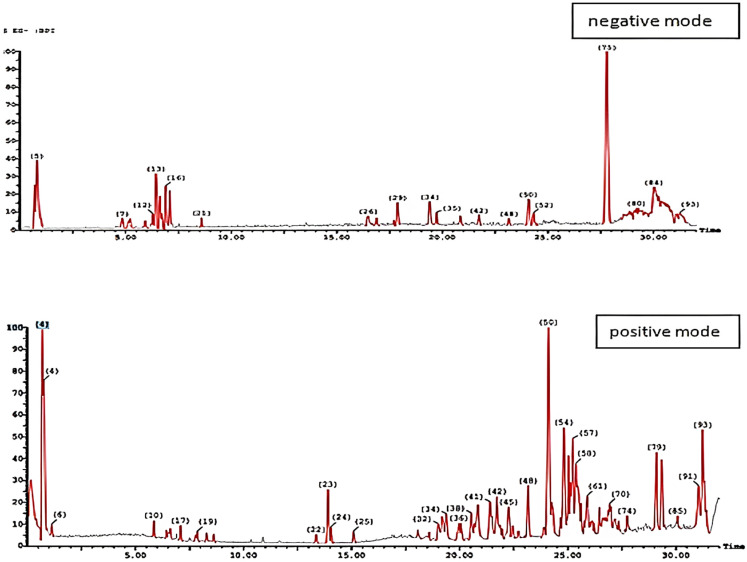
UPLC—ESI—MS/MS chromatograms of aqueous leaf capsule extract of *A. muricata* in both positive and negative ionization modes.

In the current study, HPLC–ESI-MS/MS analyses in both positive and negative ionization modes were used to perform a study of the phytochemical composition of the aqueous leaf capsule extract (Figure 1). These constituents were identified by comparing the retention times(RT) and fragmentation patterns with the reported literature.

From the aqueous leaf capsule extract of *A. muricata*, 410 components have been tentatively identified, as shown in Tables S1–S5. Among these 410 components, 384 phytochemicals were previously characterized in the fruit extracts of the same plant, as shown in Tables S1–S5 [6]. The other 26 compounds were identified exclusively from the aqueous leaf capsule extract and not reported in the fruit extract, as illustrated in Table 1. These compounds belong to variable classes, including acetogenins (eight compounds), phenolic compounds (six compounds), flavonoids (six compounds), and alkaloids (four compounds), in addition to two miscellaneous compounds. The eight acetogenins were characterized as cohibins A/B [4,7], desacetyl uvaricin [8], muricin I [4,7], 5-cis-reticulatacin-10-one [4,7], muricin D [4], muricin E [4], solamin [4], and panatellin [4] (Table 1 and Table S1, Figure S1). The six phenolic compounds include sanguisorbic acid dilactone [9], galloyl-quinic acid-rutinoside [10], mucic acid-kaempferol-malic acid-rhamnose [10], galloyl pyrogallol derivative [10], digallic acid derivative [10], catechin derivative, and caffeic acid derivative [11] (Table 1 and Table S2, Figure S1), and the six flavonoids include cyanidin-acetylglucoside pyruvic acid [12], chrysoeriol-7-*O*-glucouronyl-glucouronic acid [13], quercetin-rhmnose-sophoroside [4], kaempferol-3-*O*-glucose-rhamnose-glucoside [4], tangeretin derivative [14], and visdulin III derivative [15] (Table 1 and Table S3, Figure S1). Four alkaloids, squamolone [8], isopiline [16], vinblastine [17], and stepharine [4,14]), are also included (Table 1 and Table S4, Figure S1), along with one lignan (phillygenin-O-hexose-O-pentose) [18] and one triterpenoid (2,3,19,23-tetra-hydroxy-urs-12-en-28-oic-acid-glucose [19]) (Table 1 and Table S5, Figure S1). Conversely, the aqueous leaf capsule extract did not include the four acetogenins, named muridienin-1, muridienin-3, muridienin-4, and muricadienin, that we had previously identified (Table S1) [6].

**Figure 2:**
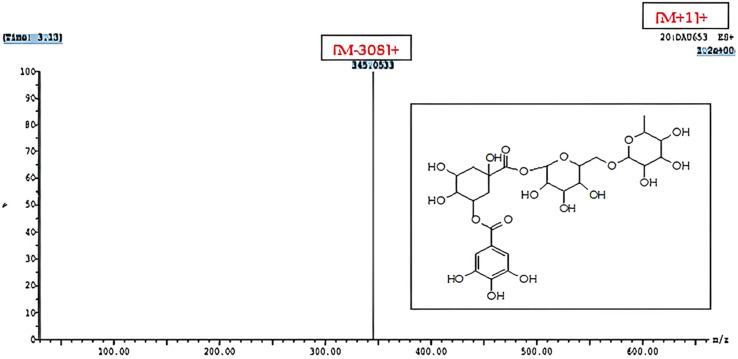
UPLC–ESI-MS/MS chromatogram of the suggested new phenolic compound detected in *A. muricata* aqueous leave capsule extract in positive (+) ionization mode.

Notably, a phenolic compound that had not previously been described from nature was tentatively identified. This compound is characterized as galloyl-quinic acid-rutinoside (Rt. 0.91 min) (Figure 2), where a molecular ion peak at *m*/*z* 653 [M+H]+ and an ion fragment at *m*/*z* 345 [M-308]+, representing the base peak, were visible in the ESI–MS/MS spectrum (Figure 2), indicating the presence of galloyl-quinic acid following the loss of the rutinoside moiety [10]. Further spectroscopic analysis should be performed to confirm the suggested structure.

### 2.2. Molecular Findings

In the current investigation, as indicated by Table 2, when Ehrlich tumor cells induce cancer, there is an increase in the expression of the gene level of Bcl-2 and a decrease in the Bax and caspase-3 genes expression. However, treatment with aqueous leaf capsule extract led to an increase in the expression of the Bax and caspase-3 genes and a decrease in the expression of the Bcl-2 gene. These results are consistent with the findings from the previous study [6], which found that the treatment of EAC mice with different *A. muricata* extracts, to varying degrees, depending on the variation in their chemical constituents, significantly ameliorated the resulting abnormalities in the gene expression of Bax and caspase-3pro-apoptotic genes and Bcl-2anti-apoptotic genes. This was primarily because of acetogenins—the main components of graviola—which induce apoptosis by increased BAXmRNA expression, causing caspase-3 to be activated due tothe release of mitochondrial cytochrome c, which forms the apoptosome complex [20].

### 2.3. Histopathological Examination

**Figure 3:**
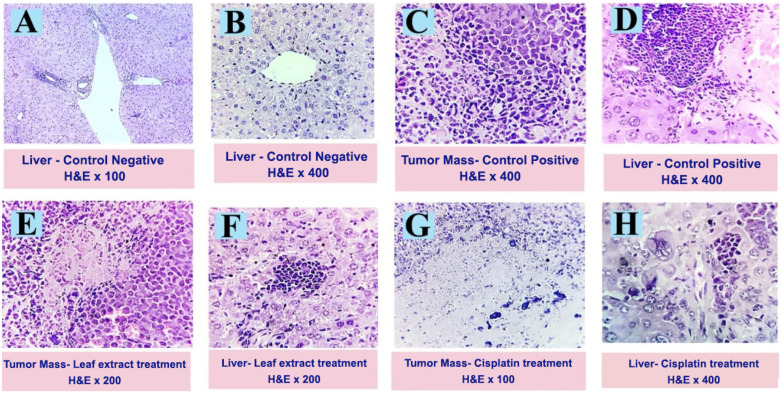
Photomicrograph of the recorded histopathological changes in the tumor mass and hepatic tissues of different experimental groups.

The current study investigated therapy regimens through immunohistochemical, morphometric, and histopathological studies. The histopathologically investigated EAC tumor mass and potential hepatic metastatic cells were noted, together with the corresponding response of immune cells and any apoptotic, necrotic, or degenerative alterations in the various experimental groups. The proliferation index, nuclear hyperchromacia, andmitotic patterns were also evaluated. Ehrlich carcinoma tumor cells replaced the peritoneal tissues and appeared as sheets, cords, clusters, and individual cells (Figure 3C). Certain tumor cells exhibit localized necrosis and apoptosis, similar to those that are observed invading the liver tissues. The EAC-bearing mice that received cisplatin treatment displayed full necrotic alterations along with focal calcification. Sections of liver tissue that were examined (Figure 3H) revealed the remains of tumor cells that had either apoptotic changes or were degraded due to an intense inflammatory response by macrophage and lymphocyte cells. In addition to the biliary proliferative alteration, the residual hepatic parenchyma showed extensive peri-portal and interstitial aggregation of inflammatory cells. When the tumor mass shrank after being treated with the leaf extract, 85–90% of the cells (Figure 3E) showed marked necrotic and apoptotic alterations. This indicated a very good ameliorative impact. Typical mitotic activity and aberrant morphologic alterations were seen in the remaining cells. The hepatic parenchyma in liver tissue sections was normal, and there were no tumor deposits. The aforementioned observations were consistent with the findings of Samin et al. (2016) and Alzergy et al. (2018) [21,22].

In Figure 3A,B (liver-control negative) control mice free from any tumor deposits in their liver tissue are shown. Ehrlich carcinoma tumor cells appear, replacing the peritoneal tissues as sheets, cords, clusters, and individual cells in Figure 3C (tumor mass-control positive). The hepatic tissue appears to show an intense inflammatory reaction in Figure 3D (liver-control positive). The EAC-bearing mice that received cisplatin treatment displayed full necrotic alterations along with focal calcification (Figure 3G) (tumor mass-cisplatin treatment). Sections of liver tissue (Figure 3H) (liver-cisplatin treatment) show the remains of tumor cells that had either apoptotic changes or degraded due to an intense inflammatory response by macrophage and lymphocyte cells. When the tumor mass shrank after being treated with the leaf extract, 85%–90% of the cells (Figure 3E) (tumor mass-leaf extract treatment) showed marked necrotic and apoptotic alterations. The liver tissue sections appeared normal, and there were no tumor deposits (Figure 3F) (liver-leaf extract treatment). H&E × 100, 400.

**Figure 4:**
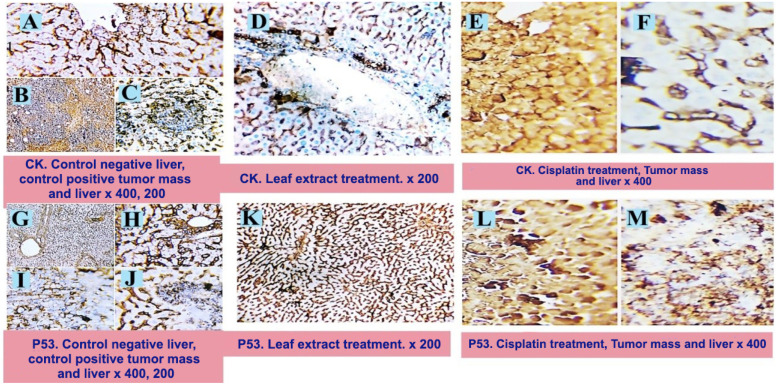
Photomicrograph of the recorded immunohistochemical changes in the tumor mass and hepatic tissues of different experimental groups showing tumor-free liver (**A**). Sections of the tumor mass(EAC), immunohistochemically stained with pan-cytokeratin(CK), clearly show the malignant cells with a noticeable brownish cytoplasmic stainability(**B**) and tumor cell deposits with moderate cytoplasmic stainability in the liver (**C**). Sections from the tumor mass of rats treated with cisplatin (**E**) show a large amount of tumor cell necrosis and a negative staining reaction; apart from a few cells that display a weak positive reaction, the liver appears free of any CK-positive tumor cells (**F**). Sections from the liver of the leaf extract treatment show no signs of metastatic or apoptotic cells (**D**). ×200, 400.

Sections of the tumor mass, immunohistochemically stained with pan-cytokeratin, underwent immunohistochemical analysis, which clearly showed that the malignant cells had a noticeable brownish cytoplasmic stainability. Figure 4B (control positive tumor mass) shows that every tumor cell tested positive for CK. Tumor cell deposits with moderate cytoplasmic stainability were seen in the liver (Figure 4C (control positive-liver) and Table 3). Sections from the tumor mass of rats treated with cisplatin (Figure 4E and Table 3) showed a large amount of tumor cell necrosis and a negative staining reaction. Hazardous brownish staining of the cytoplasmic membrane was observed in several cells. With the exception of a few sections that displayed focal aggregates of weak, positive, malignant cells, the majority of the liver sections (Figure 4F and Table 3) were clear of malignant deposits. Sections from the leaf capsule extract treatment group’s tumor mass showed significant necrosis and an erratic weak staining reaction. In contrast, sections from the group’s liver revealed peri-portal cytokeratin-stained cells with noticeable disorganization and degeneration. On the other hand, other hepatic parenchyma showed no signs of metastatic or apoptotic cells (Figure 4D and Table 3). The information provided above was consistent with the findings of Abd El-Kaream et al. (2019) and Shukry et al. (2020) [23,24].

Sections of the tumor mass that were stained for apoptotic factor P53 revealed a significant percentage of cells that did not react well to the stain. Figure 4H shows the appearance of a few apoptotic tumor cells with a dark, positive, dangerous cytoplasmic staining reaction. Liver sections exhibited cancerous cell formations with nearly non-existent staining responses to the used marker. Only a small percentage of cells responded randomly to the applied marker (Figure 4J and Table 3). Large numbers of necrotic cells with a hazardous staining response to the marker employed were seen in sections from the tumor mass that had received cisplatin treatment (Figure 4L and Table 3). The apoptotic response was seen in certain cells, as indicated by dark, positive, brownish nuclear staining. The liver had necrotic deposits as well, and some of the cells had apoptotic-looking nuclear and cytoplasmic staining reactions(Figure 4M and Table 3). Treatment with the leaf capsule extract indicated that no positively labeled apoptotic cells or metastatic tumor cells were present (Figure 4K and Table 3). These results are in agreement with those reported in Prasad et al. (2019) [25]. The authors indicated that the extract from the capsules has a positive effect on patients’ health. It contains extracted, purified active compounds that can be used with other chemical treatments to combat the highly social battle of life (tumor spread and death).

The results of this study may be consistent with our earlier study [6], which demonstrated that different extracts showed variable anticancer activities. This can be explained by the variability in bioactive compounds present in different plant extracts, as previously mentioned in this study. The different extracts showed the same constituents, with some variable constituents between the three extracts. These were mostly phenolic compounds famous for their antioxidant activity, which exerts a synergistic effect of anticancer properties.

Sections of the tumor mass that were stained for apoptotic factor P53 show apoptotic tumor-free hepatic parenchyma (Figure 4G,H). The EAC (Figure 4I) shows the appearance of a few apoptotic tumor cells with a dark, positive, dangerous cytoplasmic staining reaction. Liver sections exhibited cancerous cell formations with nearly non-existent staining responses to the used marker. Only a small percentage of cells responded randomly to the applied marker (Figure 4J). Large numbers of necrotic cells with a hazardous staining response to the marker employed are seen in sections from the tumor mass that had received cisplatin treatment (Figure 4L). The apoptotic response was seen in certain cells, as indicated by dark, positive, brownish nuclear staining. The liver shows necrotic deposits as well, and some of the cells with apoptotic-looking nuclear and cytoplasmic staining reactions (Figure 4M). Treatment with the leaf extract indicated that no positively labeled apoptotic cells or metastatic tumor cells were present (Figure 4K). ×200, 400.

Our study agreed with the apoptotic effects found in vivo, where *A. muricata* inhibited the progression of orthotopically implanted breast and pancreatic tumors in mice and chemically induced breast cancer in rats [20].

However, Zeweil et al. (2019) [20] reported mitochondrial-dependent apoptotic pathways, proving that graviola is a powerful anticancer agent that does not cause additional harm to normal cells. This indicates the selectivity of graviola on cancer cells and elicits the safety of graviola on animals, unlike conventional anticancer drugs that show severe toxicity [20]. Moghadamtousi et al. (2015) [4] also reported that graviola induced apoptosis by activating caspases 3/7 and 9, upregulating Bax, and downregulating Bcl-2 at the mRNA and protein levels.

## 3. Materials and Methods

### 3.1. Plant Materials and Extract Preparation

Graviola leaves extract supplement capsules were bought in November 2020 from the market. The graviola extraction process took place at Zagazig University’s Faculty of Pharmacy. The contents were freshly dissolved in distilled water before being administered, with four capsules containing six grams of powder dissolved in 300 milliliters of distilled water [22].

### 3.2. Analysis of A. muricata Extracts Using UPLC-ESI-MS/MS

#### 3.2.1. Separation Technique and LC/MS Instrument Conditions and Parameters

After filtering with an LC-MS syringe membrane filter of a pore size of 0.2 µm, the sample solutions of an aqueous leaf extract from the capsules prepared at a concentration of 100 µg/mL were produced using MeOH solvent of HPLC analytical grade and then subjected to analysis by LC-ESI-MS. The ultraperformance liquid chromatography (UPLC) equipment of a model called XEVO-TQD triple quadruple, purchased from Waters Corporation, located in Milford, MA, USA, was used to inject samples with injection volumes of 10 µL. It was fitted with a reversed-phase C-18 column, named ACQUITY UPLC-BEH C18, with the following dimensions: 2.1 × 50 mm and 1.7 µm. Prior to injection, the mobile phase was degassed by sonication and prepared by filtering through a 0.2 µm MS membrane disk filter. A gradient elution was used with a mobile phase made up of two solvents—solvent A was H_2_O, acidified with formic acid (0.1%), and solvent B was MeOH, acidified with formic acid (0.1%)—and was used to achieve mobile phase elution and separation. The flow rate was set at 0.2 mL/min. The gradient used for elution was 20% B, 0–1 min; 20–90% B, 1–18 min; and 20% B, 18–20 min. The following values were utilized for the analysis parameters: 150 °C for the source temperature; the cone voltage was set at 30 eV; the capillary voltage was adjusted at 3 kV, which was in addition to the temperature of desolvation at450 °C; the gas flow of the cone was at 50 L/h; and the desolvation gas flow was at 900 L/h. These values were used in both the negative and positive ion modes.

#### 3.2.2. UPLC-ESI-MS-MS Analysis

Between 50 *m*/*z* and 900 *m*/*z*, the mass spectra of the detected compounds were found in the ESI negative and/or positive ion modes. Maslynx 4.1 software was used to process the peaks and spectra, and the retention time (Rt) and mass spectrum of the peaks and spectra were compared with the reported data to make a preliminary identification. A measure of 40 eV was employed as the fragmentation collision energy.

### 3.3. Cytotoxic Activity

The Zagazig University Scientific and Medical Research Center (ZSMRC) hosted the cytotoxic study. Forty mice were used in the experiment, split into four groups of ten mice each at random.

#### 3.3.1. Experimental Animals

Forty Swiss albino mice of male sex with weights of between 25 and 30 *g* were procured from the National Cancer Institute’s animal house situated in NCI at Cairo University of Egypt. The animals were maintained in typical laboratory settings, with aeration, and the room temperature was at around 25 °C inside the metal cages. They had access to enough water and food for rodents. The Institutional Animal Care and Use Committee(IACUC) released the approval number ZU-IACUC/2/F/91/2020 at the Zagazig University research center. In adherence to the ethical standards, the animals were subjected to treatment and subsequently sacrificed based on the provided approval.

#### 3.3.2. Ehrlich Ascites Carcinoma (EAC)

The in vivo EAC cells were obtained from Swiss albino male mice from the National Cancer Institute (NCI) located at Cairo University (Egypt).

#### 3.3.3. Cisplatin

Cisplatin was used as a positive control anticancer medication, and it was obtained from Mylan Pharma in France. The cisplatin standard was freshly prepared before each treatment.

#### 3.3.4. Cancer Induction by Ehrlich Ascites Carcinoma

The mice in Groups II–IV(GII-GIV) (n = 30) had their right thighs of the lower limb subcutaneously injected with 2.5 × 106 cells to start the solid tumors. A week later, the tumors appeared [26].

#### 3.3.5. Treatment Regimen

For 28 days, Group I, which included healthy mice, was simply given the standard laboratory diet and tap water. Only the EAC cells were given to Group II, which served as the positive control. Group III, the cisplatin-treated group, received weekly doses of cisplatin at a dose of 2 mg/kg I.P. following ten days of induction EAC for 28 days on days 10, 17, and 24 [27]. After ten days of inducing EAC for 28 days, Group IV (aqueous leaf capsule extract-treated group) was given a water extract of leaf capsule orally at a dose of 200 mg/kg each day [28].

#### 3.3.6. Tissue Samples

The animals were sacrificed and dissected right away after their blood was drawn. After removing the tumor mass and liver, they were cleaned of any remaining blood with 0.9% NaCl and dried on paper. To ascertain the gene expression of Bax, Bcl-2, and caspase-3 via the reverse transcription polymerase chain reaction (RT-PCR), portions of the tumor masses in each group were stored at −80 °C. For histological and immunohistochemical analyses, slices of the tumor mass and other liver sections were preserved in 10% neutral buffered formalin.

#### 3.3.7. Molecular Determination

Using an RNA extraction kit obtained from Thermo Fisher Scientific, Inc. (Dreieich, Germany), total RNA was extracted from the tumor mass. Using the HiSenScriptTM RH (-) c DNA Synthesis kit provided by iNtRON Biotechnology Co. (Seongnam, Kyonggi-do, Republic of Korea), total RNA was reverse transcribed to complementary DNA (cDNA) in a Veriti 96-well thermal cycler obtained from Applied Biosystems (Foster City, CA, USA) for 60 min at 45 °C and then for 10 min at 85 °C. For the PCR, the primer sequence (50–30) was as follows: Bax (forward primer: CTACAGGGTTTCATCCAG and reverse primer CCAGTTCATCTCCAATTCG); Bcl-2 (forward primer: GTGGATGACTGAGTACCT and reverse primer CCAGGAGAAATCAAACAGAG); caspase-3 (forward primer TGCGTGTGGAGTATTTGGATG and reverse primer TGGTACAGTCAGAGCCAACCTC); and GAPDH (forward primer GAGAAACCTGCCAAGTATG and reverse primer GGAGTTGCTGTTGAAGTC). The thermal cycling settings were one cycle for denaturation at 95 °C for 12 min, forty cycles for 15 s at 95 °C, 30 sat 60 °C for annealing, and extensions for 30 sec at 72 °C. The relative CT approach was used for all samples for comparison.

#### 3.3.8. Histopathological Examination

Different mice groups’ tumor masses and livers were promptly excised, and the tissue was preserved in neutral buffered formalin (10%) for histological and immunohistochemical analyses. The tissue was fixed by immersing it in buffered formalin(10%) for 48 h, and then the fixative was removed for 30 min using distilled water. The tissue was then subjected to alcohol (70%) for 2 h, then alcohol (90%) for 1.5h, and was followed by two cycles of 100% alcohol, each lasting an hour, in order to dehydrate it. The samples were then cleaned using multiple xylene changes. This involved immersing the tissue for an hour in an alcohol/xylene (1:1) mixture, then for 1.5 hin pure xylene. After being impregnated with melted paraffin wax, the samples were blocked out and embedded. Hematoxylin and Eosin staining was applied to the paraffin sections (4–5 µm) [29]. The stained sections were inspected for any pathological alterations, necrosis, apoptosis, degeneration, inflammation, and abnormalities of the circulatory system.

#### 3.3.9. Immunohistochemistry Investigation

Microwave treatment was applied to the tissue sections. The two-step immunostaining method was used to determine whether antigens were present in the tissues. A biotin–streptavidin (BSA) system was used to visualize the reaction once the primary antibody had been linked to the relevant antigen [30]. The current experiments used 3,30 Hematoxylinfor counterstaining, and the permanent preparation was conducted using diaminobenzidine (DAB). Positively charged glass slides, purchased from Biogenex, Freemont, CA, USA, were used to mount the paraffin sections that were five microns thick. Following an overnight soak in xylene, the paraffin sections were run through ethanol at 50%, 75%, 95%, and 100% concentrations. The slides were dried after any extra buffer was wiped off. The sections were covered with a single drop of supersensitive primary monoclonal antibodies (Pan-Cytokeratin and P53). The slides were incubated for 60 min and then washed in phosphate-buffered saline (PBS) for 5 min. After applying two drops of DAKO EnVision for twenty min, the PBS was used for rinsing. After applying DAB chromogen for 10 to 20 min to achieve the required brown color, the slides were cleaned in the buffer to remove the DAB. The nuclei in the slices were counterstained with Mayer’s hematoxylin (Hx). The sections were immersed in Hx solution for three to five min, depending on the level of nuclear staining. They were then rinsed with tap water, separated in acid–alcohol, and then rinsed again with tap water. Canadian balsam was used to mount the air-dried slides.

The slides were heated in a pressure cooker filled with Tris-buffered saline containing Tween-20 (0.075%, pH 7.6) for ten min to remove the antigen for myeloperoxidase immunohistochemistry [31]. After that, the samples were incubated for 20 minat room temperature in 0.3% *v*/*v* H_2_O_2_ in methanol [32]. This was conducted to decrease the endogenous peroxidase activity. The sections were stained after being incubated at room temperature for 30 min with a 1:1500 dilution of the polyclonal rabbit antihuman myeloperoxidase antibody. Using an avidin–biotin–horseradish peroxidase system provided by Vector Laboratories (Burlingame, CA, USA) for the myeloperoxidase and diaminobenzidine obtained from Kirkegaard and Perry Laboratories (Gaithersburg, MD, USA) for CD68, immunostaining was carried out.

#### 3.3.10. Morphometric Analysis

A 40× objective and an Olympus digital camera, provided by Olympus LC20 (Tokyo, Japan), which was mounted on an Olympus microscope produced by Olympus BX-50 (Tokyo, Japan) with a 1/2× photo adaptor were used to digitize the image analysis slides. Using Video Test Morphology 5.2 software, provided by Moscow, Russia, which has a special built-in routine for immunohistostaining analysis and stain quantification, the resultant images were examined on an Intel^®^ Core I3^®^-powered computer. The area % of positive expression for caspase-3 was measured by the system. Each tissue was imaged five times, 200 µm apart. To evaluate the positive cells, five views per slice were selected at random and subjected to the image analysis software (Microvisioneer, Ledererzeile 31, 83512 Wasserburg am Inn, Germany, https://www.microvisioneer.com/mvslide (accessed on 6 May 2024)). An automatic calculation was made to determine the positive cells’ average grayscale [33].

### 3.4. Statistical Analysis

The mean values ± SEM (standard error of the mean) were used to express the results. Using a one-way analysis of variance (ANOVA), the impact of the treatment groups on the various biochemical markers was evaluated. The Statistical Package for Social Sciences, version 28.0 of SPSS, IBM Corp., Armonk, NY, USA, was used to conduct all analyses and graphs.

## 4. Conclusions

In a pharmaceutical landscape, plants with a long history of use in ethno-medicine are a rich source of active phytoconstituents that provide medicinal or health benefits against various ailments and diseases.

The current work examined the underlying processes and anticancer properties of *A. muricata* aqueous leaf extract from capsules in an animal model and profiled the secondary metabolites in the extract. Using HPLC-ESI-MS/MS, approximately 410 chemicals—classified as alkaloids, flavonoids, tannins, phenolics, and acetogenins (the main classes of active phytoconstituents)—were tentatively identified. Among them, one novel component was discovered for the first time in nature, and 26 phytoconstituents were exclusively identified from the aqueous leaf capsule extract. Promising anticancer activities were obtained in all the in vitro assays and in a cell-based model against the deleterious effects of EAC when comparing the current study with our previous study on the ethanolic extract of the whole fruits and the aqueous extract of the edible fruit part. The harmful effects of EAC in rats were mitigated by the extract. The present study’s LC-MS spectrum identified high-content phenolic compounds in *A. muricata*, which were tentatively characterized for the first time. The cytotoxic effects may be related to its high content of polyphenolic compounds. These extracts’ broad range of phytoconstituents may have contributed to their potency, as seen by their cytotoxic effects. The differences in activity between the various extracts were not statistically significant enough to rule out the possibility of using any portion of the plant accessible to us as a natural source of cytotoxic therapy.

In conclusion, these data are considered an addition to the bibliographic data about *A. muricata* and are a contribution towards the exploration of its chemical diversity as well as nutritional and therapeutic values. Henceforth, further studies should be focused on the isolation of the active principles and studying their efficacy, exact mechanisms, and safety profile, which could aid in the development of a new therapeutic agent and safe, natural alternative therapies for the treatment of different diseases.

Further phytochemical and pharmacological studies should be conducted to elucidate the chemical structure of the newly and tentatively identified secondary metabolite using extensive spectroscopic and spectrometric methods. Additionally, bioassay-guided fractionation for the bioactive extract should be considered to pursue the bioactivity and isolate the bioactive compounds.

## Figures and Tables

**Table 1:** Phytochemical compounds detected and characterized in *A. muricata* aqueous extract of the leaves capsuleusing HPLC–ESI-MS/MS in positive and negative ionization modes.

No	Cpd-Name	Type	Rt	Mwt	M±	Ms/MsFragment	Ref.
**1**	**^🏶🏶^ Sanguisorbic acid dilactone**	**phenolic**	**0.74**	**470**	**471**	**469,314,301,286**	**[9]**
**2**	**✓ galloyl-quinic acid-rutinoside**	**phenolic**	**0.91**	**652**	**653**	**563,345,308**	**[10]**
3	**^🏶🏶^ mucic acid-kaempferol-malic acid-rhamnose**	**phenolic**	**3.13**	**740**	**739**	**739,547,192**	**[10]**
4	**^🏶🏶^ squamolone**	**alkaliod**	**4.41**	**128**	**129**	**129,112**	**[8]**
5	**^🏶🏶^ galloyl pyrogallol drv.**	**phenolic**	**4.43**	**358**	**357**	**358,277**	**[10]**
6	**^🏶🏶^ cyanidin-acetylglucoside pyruvic acid**	**flavonoid**	**6.61**	**559**	**558**	**558,359**	**[12]**
7	**^🏶🏶^ chrysoeriol-7-O-glucouronyl-glucouronic acid**	**flavonoid**	**7.07**	**652**	**653**	**653,602,351,301**	**[13]**
8	**^🏶🏶^ digallic acid drv.**	**phenolic**	**7.17**	**378**	**377**	**377,322(100%)**	**[10]**
**9**	**^🏶^ catechin drv.+caffeic acid drv.**	**phenolic**	**7.30**	**470**	**471**	**471,289,179,135**	**[11]**
**10**	**^🏶🏶^ isopiline**	**alkaliod**	**8.78**	**297**	**298**	**297,265**	**[16]**
**11**	**^🏶^ vinblastine**	**alkaliod**	**10.29**	**811**	**810**	**810**	**[17]**
12	**^🏶^ quercetin-rhamnose-sophoroside**	**flavonoid**	**11.70**	**756**	**757**	**757,308,302,146**	**[4]**
13	**^🏶^ kaempferol-3-O-glucose-rhamnose-glucoside**	**flavonoid**	**11.81**	**756**	**757**	**755,448,470**	**[4]**
14	**^🏶🏶^ phillygenin-O-hexose-O-pentose**	**lignan**	**17.12**	**666**	**667**	**667,373,534**	**[18]**
**15**	**^🏶🏶^ Tangeretin-drv.**	**flavonoid**	**17.53**	**740**	**739**	**739,371**	**[14]**
**16**	**^🏶🏶^ visIdulin III-drv.**	**flavonoid**	**17.60**	**740**	**739**	**739,345**	**[15]**
17	**^🏶^ Cohibins A/B**	**acetogenin**	**17.71**	**548**	**547**	**548**	**[4,7]**
**18**	**^🏶^ stepharine**	**alkaloid**	**18.15**	**297**	**298**	**297,146**	**[1,4]**
19	**^🏶^ Desacetyl uvaricin**	**acetogenin**	**19.77**	**606**	**607**	**607,571,553**	**[8]**
20	**^🏶^ Muricin I**	**acetogenin**	**19.95**	**606**	**607**	**607,571,553**	**[4,7]**
21	**^🏶^ 5-Cis-reticulatacin-10-one**	**acetogenin**	**19.97**	**606**	**607**	**607,571,553**	**[4,7]**
22	**^🏶🏶🏶^ 2,3,19,23-tetra-OH-urs-12en-28oic-acid-glucose**	**triterpene**	**20.17**	**666**	**667**	**667,503,162**	**[19]**
23	**^🏶^ Muricin D**	**acetogenin**	**22.44**	**568**	**569**	**569,533**	**[4]**
**24**	**^🏶^ Muricin E**	**acetogenin**	**23.02**	**568**	**569**	**569,533**	**[4]**
**25**	**^🏶^ Solamin**	**acetogenin**	**25.77**	**564**	**563**	**563**	**[4]**
**26**	**^🏶^ Panatellin**	**acetogenin**	**28.58**	**564**	**563**	**563**	**[4]**

✓ New compound identified in *A. muricata*; ^🏶^ compounds previously identified in *A. muricata*; ^🏶🏶^ compounds identified for the first time in *A. muricata*; ^🏶🏶🏶^ compounds identified for the first time in the *Annona* genus.

**Table 2:** The impacts of 200 mg/kg of *A. muricata* aqueous extract of the leaves capsule and 2 mg/kg of cisplatin on the expression of pro- and anti-apoptotic genes in tumor masses by real-time PCR.

Parameters	GII (EAC)	GIII (EAC + Cisplatin)	GIV (EAC + Capsule)
BAX	1.00 ± 0.43	6.08 ± 1.33 ^b^	1.95 ± 0.16
Bcl-2	1.00 ± 0.17	0.41 ± 0.11 ^b^	0.48 ± 0.14 ^b^
Casp-3	1.00 ± 0.32	5.91 ± 1.00 ^b^	1.55 ± 0.52

Results were expressed as mean ± SEM (n = 10): *p* < 0.01 for the control group, ^b^ *p* < 0.001 forthe EAC group, and *p* < 0.001 for the EAC + capsule group.

**Table 3:** Morphometric analyses of P53 and CK.

	Parameters	P^53^	CK
Groups			
Control −ve (GI)		1.68 ± 0.27	1.93 ± 0.36
Control +ve(GII)		4.50 ± 0.49 *	41.69 ± 4.95 *
Cisplatin		19.35 ± 0.44 ^$b^	17.66 ± 0.59 ^$a^
Capsule		3.30 ± 0.12 *^#^	2.65 ± 0.16 ^a#^

The findings are shown as mean ± SEM. * means significant difference from group I at *p* < 0.05; ^$^ means significant difference from group I at *p* < 0.001; ^a^ significantly different from group II (*p* < 0.05); ^b^ significantly different from group II (*p* < 0.001); ^#^ significantly different from cisplatin (*p* < 0.001).

## Data Availability

Data are contained within the article.

## Supplementary Materials

The following supporting information can be downloaded at: https://www.mdpi.com/article/10.3390/ph17050614/s1, Table S1: Phytochemical compounds (AGEs) detected and characterized in *A. muricata* in the ethanolic extract of fruit, water extract of the edible part of the fruit & aqueous extract of the leaves capsule by using HPLC–ESI-MS/MS in positive and negative ionization modes; Table S2: Phytochemical compounds (Pls) detected and characterized in *A. muricata* in the ethanolic extract of fruit, water extract of the edible part of the fruit & aqueous extract of the leaves capsule by using HPLC–ESI-MS/MS in positive and negative ionization modes; Table S3: Phytochemical compounds (Fls) detected and characterized *A. muricata* in the ethanolic extract of fruit, water extract of the edible part of the fruit & aqueous extract of the leaves capsule by using HPLC–ESI-MS/MS in positive and negative ionization modes; Table S4: Phytochemical compounds(Alks) detected and characterized *A. muricata* in the ethanolic extract of fruit, water extract of the edible part of the fruit & aqueous extract of the leaves capsule by using HPLC–ESI-MS/MS in positive and negative ionization modes; Table S5: Phytochemical compounds (miscellaneous) detected and characterized *A. muricata* in the ethanolic extract of fruit, water extract of the edible part of the fruit & aqueous extract of the leaves capsule by using HPLC–ESI-MS/MS in positive and negative ionization modes; Figure S1: Some compounds characterized from *A. muricata* aqueous leaves capsules extract; Figure S2: Photomicrograph of the recorded histopathological changes in the tumor mass and hepatic tissues of different experimental groups; Figure S3: Photomicrograph of the recorded immuohistopathological changes in the tumor mass and hepatic tissues of different experimental groups.
