# Supplementary material for: Comprehensive Chemical Profiling and Mechanistic Insight into Anticancer Activity of Annona muricata Leaves Extract
Source: Pharmaceuticals (Basel). 2024 May 10;17(5):614. doi: 10.3390/ph17050614 (PMC11124051; doi:10.3390/ph17050614)
Supplement: Supplementary file 1 [file pharmaceuticals-17-00614-s001.zip › pharmaceuticals-2973129-supplementary.pdf]

Comprehensive Chemical Profiling and Mechanistic Insight into Anticancer Activity of *Annona muricata* Leaves Extract

Rehab H. Abdallah [1,*], Al-sayed R. Al-Attar [2], Youssef M. Shehata [3], Doaa M. Abdel-Fattah [3], Rahnaa M. Atta [3], Omer I. Fantoukh [4] and Ahmed M. Mustafa [5]

[1] Department of Pharmacognosy, Faculty of Pharmacy, Zagazig University, Zagazig 44519, Egypt
[2] Department of Pathology, Faculty of Veterinary Medicine, Zagazig University, Zagazig 44519, Egypt; sayedattar50@gmail.com
[3] Department of Biochemistry, Faculty of Veterinary Medicine, Zagazig University, Zagazig 7120001, Egypt; rehabayman4117@gmail.com (Y.M.S.); rahnaaatta@yahoo.com (R.M.A.)
[4] Department of Pharmacognosy, College of Pharmacy, King Saud University, P.O. Box 2457, Riyadh 11451, Saudi Arabia; ofantoukh@ksu.edu.sa
[5] Chemistry Interdisciplinary Project (CHIP), School of Pharmacy, University of Camerino, Via Madonna delle Carceri, 62032 Camerino, Italy; ahmed.mustafa@unicam.it
* Correspondence: rehabhamed2000@yahoo.com

Abstract: The aqueous extract of *Annona muricata* L. leaves was thoroughly analyzed using the UPLC-MS/MS, in addition to a new approach of examination of the extract's impact on cancer of EAC(Ehrlich ascites carcinoma) in albino male mice. The aim was to investigate the diversity of the phytochemical constituents of the aqueous leaf capsule extract and their impacts on EAC as anticancer agents. The UPLC-ESI-MS/MS screening resulted in 410 tentatively identified metabolites. Among them, 384 compounds were tentatively identified in a previous study, besides a number of 26 compounds belonging to acetogenins, phenolics, flavonoids, alkaloids, and other miscellaneous compounds, which were exclusively identified in the aqueous extract of the leaf capsule. Interestingly, a new compound was tentatively characterized as galloyl-quinic acid-rutinoside. This study also demonstrated that treating EAC mice with an extract from *A. muricata* leaves significantly improved the abnormalities in the expression of pro-apoptotic (Bax and caspase-3) and anti-apoptotic (Bcl-2) genes. Furthermore, the extract showed good protection against induced Ehrlich hepatocarcinoma, according to the microscopical, histological, and immune-histochemical analyses of the liver tissues and tumor mass.

Keywords :*Annona muricata*; leaves ; UPLC-ESI-MS/MS; immunohistochemistry; molecular biology

Content

No.	Title	Pages
**Table S1**	Phytochemical compounds (AGEs) detected and characterized in *A. muricata* in the ethanolic extract of fruit , water extract of the edible part of the fruit & aqueous extract of the leaves capsule by using HPLC–ESI-MS/MS in positive and negative ionization modes.	3-8
**Table S2**	Phytochemical compounds (Pls) detected and characterized in *A. muricata* in the ethanolic extract of fruit , water extract of the edible part of the fruit & aqueous extract of the leaves capsule by using HPLC–ESI -MS/MS in positive and negative ionization modes.	9-12
**Table S3**	Phytochemical compounds (Fls) detected and characterized *A. muricata* in the ethanolic extract of fruit , water extract of the edible part of the fruit & aqueous extract of the leaves capsule by using HPLC–ESI -MS/MS in positive and negative ionization modes.	13-16
**Table S4**	Phytochemical compounds(Alks) detected and characterized *A. muricata* in the ethanolic extract of fruit , water extract of the edible part of the fruit & aqueous extract of the leaves capsule by using HPLC–ESI -MS/MS in positive and negative ionization modes.	17-18
**Table S5**	Phytochemical compounds (miscellaneous) detected and characterized *A. muricata* in the ethanolic extract of fruit , water extract of the edible part of the fruit & aqueous extract of the leaves capsule by using HPLC–ESI-MS/MS in positive and negative ionization modes.	19-20
**Figure S1**	Some compounds characterized from *A. muricata* aqueous leaves capsules extract.	21-24
**Figure S2**	Photomicrograph of the recorded histopathological changes in the tumor mass and hepatic tissues of different experimental groups	25
**Figure S3**	Photomicrograph of the recorded immunohistopathological changes in the tumor mass and hepatic tissues of different experimental groups	26-27

Table S1. Phytochemical compounds (AGEs) detected and characterized in *A. muricata* in the ethanolic extract of fruit , water extract of the edible part of the fruit & aqueous extract of the leaves capsule by using HPLC–ESI-MS/MS in positive and negative ionization modes

No	Cpd-name	Rt	Mwt	M ±	Ms/Ms Fragment	Ref.	E	W	C
1	●Muridienin-1	16.76	514	513	514	(Bermejo et al., 2005)	✓	×	×
2	●●Annofolin	16.87	253	252	252	(Jacobo-Herrera et al., 2019)	✓	✓	✓
3	●Cohebin A/B	17.71	548	547	548	(Moghadamtousi et al., 2015), (Bermejo et al., 2005)	×	×	✓
4	●Muricatacin	17.98	284	283	283	(Moghadamtousi et al., 2015)	✓	✓	✓
5	●Muricatin-C	18.06	610	609	609,577	(Zafra-Polo et al., 1996), (Bermejo et al., 2005)	✓	✓	✓
6	●Muricin-J	19.05	414	415	415	(Moghadamtousi et al., 2015)	✓	✓	✓
7	●●Squamocin-O1/O2	19.15	638	639	621,603	(Avula et al., 2018)	✓	×	✓
8	●●●Trilobalicin	19.62	610	609	609,557,539,521	(Avula et al., 2018)	✓	✓	✓
9	●Norcorydine	19.64	327	328	328,192	(Moghadamtousi et al., 2015) ,(Avula et al., 2018)	✓	✓	✓
10	●Epomuricenin A,B	19.70	530	529	529,292,152	(Moghadamtousi et al., 2015), (Bermejo et al., 2005),(Zafra-Polo et al., 1996)	✓	✓	✓
11	●Deacetyl uvaricin	19.77	606	607	607,571,553	(Avula et al., 2018)	×	×	✓
12	●Epomusenin A	19.84	558	559	558,336,299,265	(Moghadamtousi et al., 2015), (Bermejo et al., 2005).	✓	✓	✓
13	●Muricatenol	19.92	608	607	607	(Moghadamtousi et al., 2015), (Bermejo et al., 2005)	✓	✓	✓
14	●Muricin I	19.95	606	607	607,571,553	(Moghadamtousi et al., 2015),(Bermejo et al., 2005)	×	×	✓
15	●5-Cis-reticulatacin-10-one	19.97	606	607	607,571,553	(Moghadamtousi et al., 2015), (Bermejo et al., 2005)	×	×	✓
16	●Annomuricin A	19.99	612	611	611,593,575,539	(Moghadamtousi et al., 2015), (Bermejo et al., 2005)	×	✓	✓
17	●Annomuricin B	20.06	612	611	611,593,575,539	(Moghadamtousi et al., 2015), (Bermejo et al., 2005)	×	✓	✓
18	●●Squamostatin A	20.07	638	639	621,603	(Avula et al., 2018)	✓	×	✓
19	●●Uvaricin	20.08	648	649	649,613,595	(Bermejo et al., 2005)	✓	×	✓
20	●Gigantecin	20.30	638	639	621,603,533,403,391	(Champy et al., 2009), (Avula et al., 2018)	✓	×	✓
21	●Annonacin	20.46	596	597	579,561,543,525	(Moghadamtousi et al., 2015), (Avula et al., 2018)	✓	✓	✓
22	●●●Parvifloracin	20.48	610	609	609,557,539,521	(Avula et al., 2018)	✓	✓	✓

23	●Annomuricin C	20.53	612	611	611,593,575,539	(Moghadamtousi et al., 2015), (Bermejo et al., 2005)	×	✓	✓
24	●Muricatocin A	20.68	612	611	611,593,575,539	(Moghadamtousi et al., 2015), (Bermejo et al., 2005)	×	✓	✓
25	●Annonacin-10-one	20.69	594	593	577,559,541,523	(Moghadamtousi et al., 2015),(Avula et al., 2018)	✓	✓	✓
26	●●Purpurediolin	20.82	638	639	349,531	(Avula et al., 2018)	✓	×	✓
27	●Muricatocin B	20.84	612	611	611,593,575,539	(Moghadamtousi et al., 2015),(Bermejo et al., 2005)	×	✓	✓
28	●Muricatocin C	20.85	612	611	611,593,575,539	(Moghadamtousi et al., 2015) (Bermejo et al., 2005)	×	✓	✓
29	●●●Salzmanolin	20.87	654	655	655,619	(Avula et al., 2018)	✓	×	✓
30	●Iso-annonacin-10-one	20.88	594	593	577,559,541,523	(Moghadamtousi et al., 2015),(Avula et al., 2018)	✓	✓	✓
31	●●Annoglaucin	20.91	638	639	621,567,549,531	(Gomes et al., 2019) ,(Avula et al., 2018)	✓	×	✓
32	●*Cis*-annonacin-10-one	20.91	594	593	577,559,541,523	(Moghadamtousi et al., 2015),(Avula et al., 2018)	✓	✓	✓
33	●Muridienin-2	20.95	542	541	542	(Bermejo et al., 2005)	✓	×	×
34	●Muricin K	21.05	442	441	441	(Moghadamtousi et al., 2015)	×	✓	✓
35	●●10-hydroxyasmicin	21.07	638	639	621,567,549,531	(Avula et al., 2018)	✓	×	✓
36	●Muricadienin	21.39	514	513	514	(Moghadamtousi et al., 2015), (Bermejo et al., 2005)	✓	×	×
37	●Muricatalacin	21.40	612	611	611,593,575,539	(Bermejo et al., 2005)	×	✓	✓
38	●Monticristin	21.41	574	575	575,557	(Moghadamtousi et al., 2015), (Bermejo et al., 2005)	✓	×	✓
39	●Cohebin C	21.50	576	577	577,558	(Moghadamtousi et al., 2015), (Bermejo et al., 2005)	✓	✓	✓
40	●●Montanacin B/C	21.71	610	609	609,557,539,521	(Bermejo et al., 2005)	✓	✓	✓
41	●Cohebin D	21.73	576	577	577,558	(Moghadamtousi et al., 2015), (Bermejo et al., 2005)	✓	✓	✓
42	●Annomuricin E	21.75	612	611	611,593,575,539	(Bermejo et al., 2005)	×	✓	✓
43	●Muricapentocin	21.86	612	611	611,593,575,539	(Bermejo et al., 2005)	×	✓	✓
44	●2,4-*trans*-isoannonacin-10-one	21.90	594	593	577,559,541,523	(Moghadamtousi et al., 2015),(Avula et al., 2018)	✓	✓	✓
45	●Epumurinin B	21.95	532	533	531,295,277,237	(Melot et al., 2009)	✓	✓	✓
46	●Annonacin-A	22.05	596	597	579,561,543,525	(Moghadamtousi et al., 2015), (Avula et al., 2018)	✓	✓	✓
47	●●Bullatanocin	22.06	638	639	621,603,567,549	(Adesanwo et al., 2020) ,(Avula et al., 2018)	✓	×	✓
48	●● 12,15-*cis*-bullatanocin	22.08	638	639	621,603,567,549	(Adesanwo et al., 2020) ,(Avula et al., 2018)	✓	×	✓
49	●10-Hydroxytrilobacin	22.09	638	639	621,603,567	(Avula et al., 2018)	✓	×	✓

50	●Dieporeticanin	22.40	574	575	575,557	(Zafra-Polo et al., 1996),(Bermejo et al., 2005)	✓	×	✓
51	●Muricin D	22.44	568	569	569,533	(Moghadamtousi et al., 2015)	×	×	✓
52	●●Squamocin B	22.45	594	593	577,559,541,523	(Le Ven et al., 2014) ,(Bermejo et al., 2005)	✓	✓	✓
53	●●Montacin(cis)	22.62	610	609	609,557,539,521	(Bermejo et al., 2005)	✓	✓	✓
54	●●Asimitrin	22.65	638	639	621,603,567	(Avula et al., 2018)	✓	×	✓
55	●●4-Hydroxy trilobin	22.85	638	639	621,603,567	(Avula et al., 2018)	✓	×	✓
56	●● 4-Acetyl-annonacin	22.87	638	639	621,603,567	(Avula et al., 2018)	✓	×	✓
57	●●4-Acetylxylomaticin	22.90	638	639	621,603,567	(Avula et al., 2018)	✓	×	✓
58	●●Bullatalicin	23.00	638	639	527,469,329,309,241	(Rupprecht et al., 1990),(Le Ven et al., 2012), (Avula et al., 2018)	✓	×	✓
59	●●Bullatetrocin	23.01	638	639	621,603,567	(Avula et al., 2018)	✓	×	✓
60	●Muricin E	23.02	568	569	569,533	(Moghadamtousi et al., 2015)	×	×	✓
61	●*Cis*-annonacin	23.03	596	597	579,561,543,525	(Moghadamtousi et al., 2015), (Avula et al., 2018)	✓	✓	✓
62	●●Coreaheptocin A	23.16	642	641	641,607	(Gomes et al., 2019)	✓	×	✓
63	●*Cis*-goniothalamacin	23.17	596	597	484,384,308	(Moghadamtousi et al., 2015), (Bermejo et al., 2005)	✓	✓	✓
64	●Corepoxylone	23.19	560	561	651	(Moghadamtousi et al., 2015) , (Zafra-Polo et al., 1996)	✓	✓	✓
65	●*Cis*-corossolone	23.22	578	579	579,445,338,298	(Moghadamtousi et al., 2015), (Le Ven et al., 2014)	✓	×	✓
66	●Javoricin	23.25	596	597	597,579,661	(Moghadamtousi et al., 2015), (Bermejo et al., 2005)	✓	✓	✓
67	●Muridienin-3	23.43	542	541	542	(Moghadamtousi et al., 2015), (Bermejo et al., 2005)	✓	×	×
68	●Muridienin-4	23.72	542	541	542	(Moghadamtousi et al., 2015), (Bermejo et al., 2005)	✓	×	×
69	●Annonacinone(cis)	23.79	594	593	577,559,541,523	(Bonneau et al., 2017),(Avula et al., 2018)	✓	✓	✓
70	●●Annoheptocin B	23.90	672	671	671	(Gomes et al., 2019)	✓	×	✓
71	●Annocinone	23.93	594	593	577,559,541,523	(Moghadamtousi et al., 2015)	✓	✓	✓
72	●Arianacin	23.95	596	597	597,579,661	(Moghadamtousi et al., 2015), (Bermejo et al., 2005),	✓	✓	✓
73	●●Coreaheptocin B	24.07	642	641	641,607	(Gomes et al., 2019)	✓	×	✓
74	●Annoreticuin-9-one	24.08	594	593	577,559,541,523	(Moghadamtousi et al., 2015), (Ragasa et al., 2014)	✓	✓	✓
75	●*Cis*-annoreticuin	24.09	596	597	597,579,661	(Moghadamtousi et al., 2015) ,(Ragasa et al., 2014)	✓	✓	✓
76	●AnocatacinAand B	24.10	578	579	579,561,,543,525,507	(Moghadamtousi et al., 2015), (Bermejo et al., 2005)	✓	×	✓
77	●●Neoannonin (squamocin J)	24.26	578	579	579,310,270,243	(Zafra-Polo et al., 1996)	✓	×	✓
78	●Gigantetrocin A	24.28	596	597	597,579,661	(Moghadamtousi et al., 2015), (Bermejo et al., 2005),	✓	✓	✓
79	●Motrilin	24.34	622	623	510,320,390,392	(Le Ven et al., 2014),(Avula et al., 2018)	✓	✓	✓

80	●Epomusenin B	24.95	558	559	558,336,299,265	(Moghadamtousi et al., 2015), (Bermejo et al., 2005).	✓	✓	✓
81	●Annomutacin	25.02	624	623	623,607,589,553	(Moghadamtousi et al., 2015),(Avula et al., 2018)	✓	✓	✓
82	●Gigantetrocin B	25.11	596	597	597,579,661	(Moghadamtousi et al., 2015), (Bermejo et al., 2005).	✓	✓	✓
83	●●Annoheptocin A	25.15	670	671	671	(Gomes et al., 2019)	✓	×	✓
84	●Muricatetrocin A/B	25.23	596	597	597,579,661	(Moghadamtousi et al., 2015), (Bermejo et al., 2005).	✓	✓	✓
85	●●Goniotriocin	25.27	636	637	637,619	(Gomes et al., 2019)	✓	✓	✓
86	●Muricin A	25.37	596	597	597,579,661	(Moghadamtousi et al., 2015), (Bermejo et al., 2005).	✓	✓	✓
87	●● 9-oxo-asimicinone	25.48	636	637	637,619	(Champy et al., 2009)	✓	✓	✓
88	●Muricatin A	25.63	612	611	611,593,575,539	(Zafra-Polo et al., 1996) ,(Bermejo et al., 2005)	×	✓	✓
89	●Muricin B	25.70	596	597	597,579,661	(Moghadamtousi et al., 2015), (Bermejo et al., 2005).	✓	✓	✓
90	●Muricatin B	25.75	612	611	611,593,575,539	(Zafra-Polo et al., 1996) ,(Bermejo et al., 2005)	×	✓	✓
91	●Chatenaytrienin 1+2	25.76	540	539	539	(Moghadamtousi et al., 2015),(Bermejo et al., 2005)	×	✓	✓
92	●Solamin	25.77	564	563	563	(Moghadamtousi et al., 2015)	×	×	✓
93	●Muricatalin	25.86	612	611	611,593,575,539	(Bermejo et al., 2005)	×	✓	✓
94	●Annopentocin A+B	25.94	612	611	611,593,575,539	(Moghadamtousi et al., 2015) (Bermejo et al., 2005)	×	✓	✓
95	●Muricin L	25.95	442	441	441	(Moghadamtousi et al., 2015)	×	✓	✓
96	●Muricin-N	25.96	414	415	415	(Moghadamtousi et al., 2015)	✓	✓	✓
97	●Epoxymurin A,B	25.97	530	529	529,292,152	(Moghadamtousi et al., 2015)(Bermejo et al., 2005), (Zafra-Polo et al., 1996)	✓	✓	✓
98	●Sabadelin	26.13	530	529	529,292,152	(Moghadamtousi et al., 2015),((Bermejo et al., 2005)	✓	✓	✓
99	●Murihexol	26.19	614	615	614,578	(Moghadamtousi et al., 2015), (Bermejo et al., 2005).	×	✓	✓
100	●Donhexocin	26.22	614	615	614,578	(Moghadamtousi et al., 2015).	×	✓	✓
101	●●Squamocin I/K	26.23	578	579	579,561,543,507	(Zafra-Polo et al., 1996), (Gu et al., 1997b)	✓	×	✓
102	●Anohexocin	26.26	628	629	629,611,593,575	(Moghadamtousi et al., 2015), (Bermejo et al., 2005)	✓	✓	✓
103	●Squamocin	26.27	622	623	510,320,390,392	(Le Ven et al., 2014),(Jacobo-Herrera et al., 2019)	✓	✓	✓
104	●Murihexocin A	26.38	628	629	629,611,593,575	(Moghadamtousi et al., 2015), (Bermejo et al., 2005)	✓	✓	✓
105	●Murihexocin B	26.46	628	629	629,611,593,575	(Moghadamtousi et al., 2015), (Bermejo et al., 2005)	✓	✓	✓
106	●Cis-uvariamicin I	26.48	592	593	593,755	(Moghadamtousi et al., 2015), (Bermejo et al., 2005)	✓	✓	✓
107	●Cis-uvariamicin IV	26.61	592	593	593,755	(Moghadamtousi et al., 2015), (Bermejo et al., 2005)	✓	✓	✓
108	●Muricatenol	26.62	608	609	609,590,554	(Moghadamtousi et al., 2015), (Bermejo et al., 2005).	✓	✓	✓

109	•Cis-uvariamicin II	26.64	592	593	593,755	(Moghadamtousi et al., 2015), (Bermejo et al., 2005)	✓	✓	✓
110	•16,19-cis murisolin	26.72	580	581	581,527,509	(Moghadamtousi et al., 2015), (Bermejo et al., 2005).	✓	×	✓
111	•Murihexocin C	26.70	628	629	629,611,593,575	(Moghadamtousi et al., 2015), (Bermejo et al., 2005)	✓	✓	✓
112	•••Asimilobin	26.76	578	579	579,561,543,507	(Avula et al., 2018)	✓	×	✓
113	•Muricin-H	26.79	580	581	581,527,509	(Moghadamtousi et al., 2015), (Bermejo et al., 2005).	✓	×	✓
114	•Muricin C	26.86	596	597	597,579,661	(Moghadamtousi et al., 2015), (Bermejo et al., 2005).	✓	✓	✓
115	•Muricin F	26.97	594	593	577,559,541,523	(Moghadamtousi et al., 2015), (Bermejo et al., 2005)	✓	✓	✓
116	•Muricin G	27.17	594	593	577,559,541,523	(Moghadamtousi et al., 2015), (Bonneau et al., 2017)	✓	✓	✓
117	•Annomuricatin A	27.26	558	559	559	(Moghadamtousi et al., 2015).	✓	✓	✓
118	•Muricatatin	27.35	614	615	614,578	(Bermejo et al., 2005).	×	✓	✓
119	•Annomuricatin C	27.35	558	559	559	(Moghadamtousi et al., 2015)	✓	✓	✓
120	•Reticulatacin	27.36	592	593	593,755	(Moghadamtousi et al., 2015), (Bermejo et al., 2005)	✓	✓	✓
121	•Corrosolin	27.73	580	581	468,336	(Moghadamtousi et al., 2015),(Bermejo et al., 2005).	✓	×	✓
122	•Chatenaytrienin-1	27.79	512	511	511,457	(Moghadamtousi et al., 2015),(Bermejo et al., 2005).	✓	✓	✓
123	••• Isomurisolenin	27.80	578	579	579,561,543,507	(Avula et al., 2018)	✓	×	✓
124	•Muricoreacin	27.87	628	629	629,611,593,575	(Moghadamtousi et al., 2015), (Bermejo et al., 2005)	✓	✓	✓
125	••Annonisin	28.17	610	609	498,426,324	(Le Ven et al., 2014)	✓	✓	✓
126	•Panatellin	28.58	564	563	563	(Moghadamtousi et al., 2015)	×	×	✓
127	•Cis-annomontacin	28.59	624	623	623,587	(Moghadamtousi et al., 2015), (Avula et al., 2018)	✓	✓	✓
128	•Bullatalacin	28.61	622	623	510,336,408	(Le Ven et al., 2014),(Jacobo-Herrera et al., 2019)	✓	✓	✓
129	•Xylomaticin	28.87	624	623	623	(Moghadamtousi et al., 2015)	✓	✓	✓
130	•2,4(cis/trans)-10-R-annocin-A-one	28.93	596	597	597,579,661	(Moghadamtousi et al., 2015), (Bermejo et al., 2005).	✓	✓	✓
131	•Annomontacin	28.97	624	623	623,605,589	(Moghadamtousi et al., 2015)	✓	✓	✓
132	•Montancin-A	28.99	640	639	639,567,550	(Champy et al., 2009),(Avula et al., 2018)	✓	×	✓
133	•Chatenaytrienin-2	29.09	512	511	511,457	(Moghadamtousi et al., 2015).	✓	✓	✓
134	•Iso-annonacin	29.29	596	597	597,579,661	(Moghadamtousi et al., 2015), (Rupprecht et al., 1990).	✓	✓	✓
135	•Asmicin	29.33	622	623	510,320,392,390	(Le Ven et al., 2014),(Gomes et al., 2019)	✓	✓	✓
136	•Gigantetronenin	29.50	622	623	623,,605,,587,551	(Moghadamtousi et al., 2015)	✓	✓	✓
137	•Annocatalin	29.60	596	597	597,579,661	(Moghadamtousi et al., 2015).	✓	✓	✓
138	•••Asitrilobin A/C/D	30.01	624	623	623,587	(Avula et al., 2018)	✓	✓	✓
139	•2,4 cis-isoannonacin	30.04	596	597	597,579,661	(Moghadamtousi et al., 2015).	✓	✓	✓
140	•Epumurinin A	30.05	532	533	531,295,277,237	(Melot et al., 2009)	✓	✓	✓
141	••Rollidecin- C	30.07	578	579	579,409,380,309,241	(Gu et al., 1997a)	✓	×	✓

142	•Annopentocin C	30.10	612	611	611,593,575,539	(Moghadamtousi et al., 2015, Bermejo et al., 2005)	×	✓	✓
143	• Muricin M	30.11	422	441	441	(Moghadamtousi et al., 2015)	×	✓	✓
144	•Annomuricinone D	30.13	612	611	611,593,575,539	(Bermejo et al., 2005)	×	✓	✓
145	•longifolicin	30.18	580	581	527,509	(Moghadamtousi et al., 2015),(Bermejo et al., 2005).	✓	×	✓
146	••Annoglaxin	30.40	610	609	498,426,324	(Bermejo et al., 2005)	✓	✓	✓
147	•2,4 trans-isoannonacin	30.67	596	597	597,579,661	(Moghadamtousi et al., 2015).	✓	✓	✓
148	•2,4 cis-gigantetrocinone	30.83	596	597	597,579,661	(Moghadamtousi et al., 2015).	✓	✓	✓
149	••Annomontanin C	31.01	610	609	498,426,324	(Bermejo et al., 2005)	✓	✓	✓
150	•2,4 trans-gigantetrocinone	31.25	596	597	597,579,661	(Moghadamtousi et al., 2015).	✓	✓	✓

•Compounds previously identified in *Annona muricata*
••Compounds identified for the first time in *Annona muricata*
•••Compounds identified for the first time in *Annona genus*

- ♠ **E: Ethanol extract of the whole fruit , W: Water extract of the edible part of the fruit , C: Aqueous extract of the leaves capsule**
- ♠ **The yellow highlight refers to the compounds identified only in the aqueous extract of the leaves capsule**
- ♠ **The blue highlight refers to the compounds not identified in the aqueous extract of the leaves capsule**

Table S2. Phytochemical compounds (Pls) detected and characterized in *A. muricata* in the ethanolic extract of fruit , water extract of the edible part of the fruit & aqueous extract of the leaves capsule by using HPLC–ESI -MS/MS in positive and negative ionization modes

No	Cpd-name	RT	Mwt	M ±	Ms/Ms Fragment	Ref.	E	W	C
151	●●Methyl catechol	0.15	140	141	141,140,113,109	(Rini Vijayan and Raghu, 2019)	×	✓	✓
152	●●Malonyl coumaroyl quinic acid	0.27	422	421	421,146	(Marzouk et al., 2019)	✓	✓	✓
153	●●Succinyl-dicaffeoylquinic acid	0.41	616	617	617,517,103	(Abu-Reidah et al., 2015)	✓	✓	✓
154	✓ **Protochatechuic-coumaroyl quinic acid**	**0.42**	**474**	**473**	**473,338,135**	**(Tan et al., 2020)**	×	✓	✓
155	●●Trigalloyl-glucose	0.47	650	651	605,479,301,299	(Mena et al., 2012)	✓	✓	✓
156	●●Digalloyl hexose malic acid	0.70	600	601	601,303,297	(Abu-Reidah et al., 2015)	✓	×	✓
157	●●Disuccinyl-caffeoylquinic acid	0.71	554	555	555,537 ,353,191	(Lachowicz et al., 2020)	✓	×	✓
158	●Dicaffeoyl quinic acid	0.72	516	515	515,354	(Moghadamtousi et al., 2015)	✓	×	✓
159	●Feuloulcaffeoyl quinic acid	1.53	530	529	529, 180	(Moghadamtousi et al., 2015)	✓	✓	✓
160	●●Diferuloyl-syringic acid	1.70	550	551	550,354,197	(El-Hawary et al., 2020)	✓	×	✓
161	●● Sanguisorbic acid dilactone	0.74	470	471	469,314,301,286	(Lachowicz et al., 2020)	×	×	✓
162	●●Galloyl-valonic acid biloactone	0.80	662	661	469,393,169	(Abu-Reidah et al., 2015)	✓	✓	✓
163	●3-O-caffeic-quinic acid+ procyandrv.	0.82	578	577	577,289,,191,179	(Mancini et al., 2018)	✓	×	✓
164	✓ **galloyl-quinic acid-rutinoside**	0.91	**652**	**653**	**563,345,308**	**(Abu-Reidah et al., 2015)**	×	×	✓
165	●●Coumaroy lshikimic acid pentoside	1.13	452	453	453,321,132	(Tan et al., 2020)	✓	✓	✓
166	●●Di-caffeic acid	1.14	342	341	341,160,179,280	(Plazonić et al., 2009)	✓	✓	✓
167	●●Caffeoyl shikimic acid	1.15	336	337	336,174,162	(Kang et al., 2016)	✓	×	✓
168	●●Malonyl-mono CQA3	1.29	440	441	352,265,173,87	(Zhang et al., 2007)	✓	✓	✓
169	●Caffeoyl quinic acid (chlorogenic acid)	4.00	354	355	355,193	(Moghadamtousi et al., 2015), (Mancini et al., 2018)	✓	✓	✓
170	●●Abscisic acid-O-hexose-HMG	1.30	586	587	587,482	(El-Sayed et al., 2017)	×	✓	✓
171	●● Phenyl acetic acid pentoside	1.32	268	269	269,136,133	(Saftić et al., 2019)	✓	✓	✓
172	● Ferulic acid hexoside	1.33	356	355	356,193,162	(Moghadamtousi et al., 2015)	✓	✓	✓
173	●●Coumaric acid hexoside drv.	1.47	422	421	421,164	(Boukhalkhal et al., 2020)	✓	✓	✓
174	●● Caffeic acid hexose	1.52	342	341	341,179	(Rini Vijayan and Raghu, 2019)	✓	✓	✓

175	●●Ellagic acid-rhamnoside	1.70	448	449	302,228	(Mena et al., 2012)	✓	×	✓
176	●● Caffeic acid arabinose	1.71	312	313	312,179	(Oliveira-Alves et al., 2017)	✓	✓	✓
177	●●Ellagic acid-hexose	1.72	464	465	301,300	(Yisimayili et al., 2019)	×	✓	✓
178	●●Galloyl HHDP-gluconate	1.74	650	651	497,301,257,229	(Mena et al., 2012)	✓	✓	✓
179	●●Protocatechuic acid glucoside	2.01	316	315	315,153	(Jiménez-González et al., 2018)	✓	✓	✓
180	●● Ferulic acid arabinose	2.03	326	325	193,177,149,134	(Oliveira-Alves et al., 2017)	✓	✓	✓
181	●Coumaric acid glucoside	2.04	326	325	325,249,165	(Moghadamtousi et al., 2015)	✓	✓	✓
182	●●Coumaric acid-rhamnose	2.05	310	309	309,146	(Rini Vijayan and Raghu, 2019)	✓	✓	✓
183	●● Syringic acid pentoside	2.07	330	331	331,198,133	(Tan et al., 2020)	✓	✓	✓
184	●● Dimer of tergallagic acid-hexose	2.08	632	633	451,301,299	(Lantzouraki et al., 2015)	✓	×	✓
185	● Caffeic acid	2.09	180	179	179	(Coria-Téllez et al., 2019)	✓	✓	✓
186	● Quinic acid	2.28	192	193	193,173,129,113	(Larrazábal-Fuentes et al., 2020)	✓	✓	✓
187	● Gallic acid	2.46	170	169	169,152	(Moghadamtousi et al., 2015), (Coria-Téllez et al., 2019).	×	✓	✓
188	● Citric acid	2.54	192	193	104,85	(Cristofori et al., 2011)	✓	✓	✓
189	●Ellagic acid	2.69	302	303	285,275,229	(Souza et al., 2018),(Oboh et al., 2015)	✓	✓	✓
190	●●Shikimic acid hexoside	**2.75**	336	337	336,174,162	(Kang et al., 2016)	✓	×	✓
191	●●Shikimic acid	2.76	174	175	175,174(100%),131	(Marzouk et al., 2019)	✓	✓	✓
192	●●Sinapic acid drv.	2.80	436	437	437,224	(Tan et al., 2020)	✓	✓	✓
193	●● Caffeoyl-shikimic acid drv.	2.84	586	587	387,336	(Simirgiotis et al., 2017)	×	✓	✓
194	●●Caftaric acid	2.90	312	313	313,179	(Tan et al., 2020)	✓	✓	✓
195	●● Hibiscus acid	3.05	190	191	191	(Izquierdo-Vega et al., 2020)	✓	×	✓
196	●*p*-coumaric acid	3.06	164	165	164,146	(Adesanwo et al., 2020) ,(George et al., 2015)	✓	✓	✓
197	●*p*-coumaric acid-methyl ether	**3.09**	178	179	179,165	((Moghadamtousi et al., 2015)	✓	✓	✓
198	**●●mucic acid-kaempferol-malic acid-rhamnose**	**3.13**	**740**	**739**	**739,547,192**	**(Abu-Reidah et al., 2015)**	×	×	✓
199	●●Dicaffeic acid drv.	3.18	616	617	617,,341	(Rini Vijayan and Raghu, 2019)	✓	✓	✓
200	●●Hydroxyl ferulic acid drv.	3.25	318	319	319,210	(Tan et al., 2020)	✓	✓	✓
201	●● Ferulic acid drv.	3.27	273	272	272,158,132,125	(Rini Vijayan and Raghu, 2019)	✓	✓	✓
202	●●*p*-coumaric acid drv.	3.43	360	361	361,214,147,118	(Saftić et al., 2019)	✓	✓	✓

203	●● Gallic acid drv.	3.70	266	265	265,170	(Tan et al., 2020)	✓	✓	✓
204	●●Coumaric acid drv.	3.82	294	293	293,163	(Tan et al., 2020)	✓	✓	✓
205	●●Ellagic acid drv.	3.92	440	441	441,302	(Tan et al., 2020)	✓	✓	✓
206	●● Gallic acid drv.	3.93	398	399	399,171	(Tan et al., 2020)	×	✓	✓
207	●●Galloflavin	4.06	278	279	279,197	(Zhang et al., 2018)	✓	×	✓
208	●●Trigalloy llevoglucosan	4.07	618	619	619,153,109	(Abu-Reidah et al., 2015)	✓	✓	✓
209	●●galloyl pyrogallol drv.	4.43	358	357	358,277	(Abu-Reidah et al., 2015)	×	×	✓
210	●● Hibiscus acid drv.	4.44	308	309	309,172	(Tan et al., 2020)	✓	×	✓
211	●● Di-*O*-galloyl-HHDD protoquercitol I	5.09	618	619	301	(Abu-Reidah et al., 2015)	✓	✓	✓
212	●● Quinic acid drv.	5.49	330	331	331,193,175	(Tan et al., 2020)	✓	✓	✓
213	●● Maclurin-3-O-glucoside	5.77	424	425	353,341,329,287,261	(Beelders et al., 2014)	✓	×	✓
214	●● Malic acid hexose drv.	5.80	436	437	437,348,297	(Abu-Reidah et al., 2015)	✓	✓	✓
215	●● Caffeic acid drv.	6.70	378	377	377,179(100%)	(Bystrom et al., 2008)	✓	✓	✓
216	●●Maclurin drv.	6.99	360	361	361,262	(Beelders et al., 2014)	✓	✓	✓
217	●●Muclurin drv.	7.03	380	381	381,262	(Beelders et al., 2014)	✓	✓	✓
218	●● Gallic-malic acid drv.	7.07	398	399	399,286	(Abu-Reidah et al., 2015)	×	✓	✓
219	●●Hibiscus acid drv.	7.09	452	453	453,190	(Tan et al., 2020)	✓	✓	✓
220	●●digallic acid drv.	7.17	378	377	377,322(100%)	(Abu-Reidah et al., 2015)	×	×	✓
221	●● Ferulic acid drv.	7.29	382	383	383,206,149,134	(Kammerer et al., 2004)	✓	✓	✓
222	●catechin drv.+caffeic acid drv.	7.30	470	471	471,289,179,135	(Mancini et al., 2018)	×	×	✓
223	●Eugenol	7.49	164	165	165	(Adesanwo et al., 2020)	✓	✓	✓
224	●● Hydroxyl citric acid drv.	8.43	336	337	336,192	(Falcão et al., 2013)	✓	×	✓
225	●●Ellagitannin	8.80	785	786	618,302,277,251	(Nuncio-Jáuregui et al., 2015)	×	✓	✓
226	●●Brevifolin	9.30	248	249	249,219,191	(Yisimayili et al., 2019)	×	✓	✓
227	●●Caftaric acid drv.	9.38	668	669	312	(Abu-Reidah et al., 2015)	✓	✓	✓
228	●●Tetramethyl benzoic acid	10.15	178	179	179,150,122	(Adesanwo et al., 2020)	✓	✓	✓
229	●●Catechin-drv.	10.89	398	399	399,290	(Tan et al., 2020)	×	✓	✓
230	●6-(benzyloxy)-methyl-2,3,4, tri-methyl cyclohexyl formaldehyde	11.58	274	275	275	(Adesanwo et al., 2020)	✓	✓	✓
231	●Allyl pyrocatecol-diacetate	12.0	234	235	235	(Adesanwo et al., 2020)	✓	✓	✓

232	••Ellagitannin I,II	12.01	644	645	301,283,257,193	(Lantzouraki et al., 2015)	✓	×	✓
233	••9-COA	13.05	398	399	399,220,206,179,135	(Zhang et al., 2007)	×	✓	✓
234	•• Iriflophenone-3-C- glucoside	13.36	408	409	409,273,220,120	(Beelders et al., 2014)	✓	×	✓
235	••Galloylpyrogallol	13.79	278	279	279,153	(Abu-Reidah et al., 2015)	✓	×	✓
236	••Galloyl arbutin	15.17	424	425	273	(Abu-Reidah et al., 2015)	✓	×	✓
237	••Galloylpyrogallol drv.	15.49	436	437	437,279	(Abu-Reidah et al., 2015)	✓	✓	✓
238	••Methylester of lignoceric acid	19.38	382	383	383	(Ibrahim, 2012)	✓	✓	✓
239	•• Methyl corilagin	21.95	648	647	649,634,301	(Yisimayili et al., 2019)	✓	✓	✓
240	•• Dimethyl-corilagin	22.24	662	661	661,649,634,301	(Yisimayili et al., 2019)	✓	✓	✓
241	••Dihydroisovalerate	30.15	424	425	425,365,281	(Abu-Reidah et al., 2015)	✓	×	✓

•Compounds previously identified in *Annona muricata*

••Compounds identified for the first time in *Annona muricata*& annona genus

✓ New compounds identified in *Annona muricata*

HMG= 3- hydroxyl-3-methyl glutary, **CQA**= monocaffeoylquinic acid(chlorogenic acid), **COA**= caffeoyl-2,7-anhydro-2- octulopyranosonic acid

♠ E**: Ethanol extract of the whole fruit , W: Water extract of the edible part of the fruit , C: Aqueous extract of the leaves capsule**

♠ **The yellow highlight refers to the compounds identified only in the aqueous extract of the leaves capsule**

♠ **The red colored lines refers to the new compounds identified in *A. muricata* in the ethanolic extract of fruit , water extract of the edible part of the fruit & aqueous extract of the leaves capsule**

Table S3. Phytochemical compounds (Fls) detected and characterized *A. muricata* in the ethanolic extract of fruit , water extract of the edible part of the fruit & aqueous extract of the leaves capsule by using HPLC–ESI -MS/MS in positive and negative ionization modes

No	Cpd-name	Rt	Mwt	M ±	Ms/Ms Fragment	Ref.	E	W	C
242	✿✿Malonylated-luteolin-O-xylose-glucose	3.95	668	669	699,537,132	(El Sayed et al., 2016)	✓	✓	✓
243	✿✿Apigenin-pentosyl-(hydroxyferuloyl)-pentoside	6.28	726	725	533,595,325,402,271	(Benayad et al., 2014)	✓	✓	✓
244	✿✿cyanidine-acety lglucoside pyruvic acid	6.61	559	558	558,359	(He et al., 2012)	×	×	✓
245	✿✿ Luteolin-O-caffeoylglucoside	6.72	610	609	609,180	(Gu et al., 2012)	✓	✓	✓
246	✿✿Acacetin-hexose-hexose-glucouronic acid	6.75	784	785	785,608,447,338,284,240,211,176,162	(Gu et al., 2012)	✓	✓	✓
247	✿✿chrysoeriol-7-O-glucouronyl-glucouronic acid	7.07	652	653	653,602,351,301	(Marczak et al., 2016)	×	×	✓
248	✿✿ Quercetin hexose-malic acid	7.85	580	581	463,301	(Abu-Reidah et al., 2015)	✓	×	✓
249	✿✿Myricetin-galloyl-pentose	8.26	602	603	319,132,171	(Saldanha et al., 2013)	✓	×	✓
250	✿✿Myricetin-rhamnose malic acid	8.29	580	581	581,463,316,301	(Abu-Reidah et al., 2015)	✓	×	✓
251	✿✿ Kaempferol-gallic acid hexose	8.59	600	601	601,438,163	(Abdel-Hameed et al., 2013)	✓	×	✓
252	✿✿Myricetin-galloyl-hexose	9.98	632	633	317	(Abu-Reidah et al., 2015)	✓	×	✓
253	✿✿Velutin-galloyl hexoside	10.11	646	645	645,332,314	(Abu-Reidah et al., 2015), (Tan et al., 2020)	✓	✓	✓
254	✿✿Delphnidin-*p*-coumaroyl hexose	10.84	611	612	611,303	(Šuković et al., 2020)	×	✓	✓
255	**✓Dihydromyricetin galloyl hexoside**	**10.92**	**634**	**635**	**635,320,162,152**	**(Tan et al., 2020)**	✓	×	✓
256	✿✿Dihydroxy gallocatechin	11.07	342	341	341,305	(Ambigaipalan et al., 2016)	✓	✓	✓
257	**✓Apigenin-gallate**	**11.14**	**422**	**421**	**421,170,151**	**(Tan et al., 2020)**	✓	✓	✓
258	✿quercetin-rhamnose-sophoroside	11.70	756	757	757,308,302,146	(Moghadamtousi et al., 2015)	×	×	✓
259	✿kaempferol-3-O-glucose-rhamnose-glucoside	11.81	756	757	755,448,470	(Moghadamtousi et al., 2015)	×	×	✓
260	✿ Quercetin-hexoside-rhamnoside-pentose	12.07	742	743	743,308,303	(Mancini et al., 2018)	✓	✓	✓
261	✿✿Dihydrokaempferol-rhamnoside - hexose-pentose	12.25	728	727	727,288,294	(Martucci et al., 2014)	×	✓	✓

262	**✓Dihydromyricetin-hexouronic acid-hexoside**	**12.36**	**658**	**657**	**657,319,176**	**(Tan et al., 2020)**	✓	×	✓
263	●● Kaempferol/luteolin-O-pentose-O-glucouronic acid	12.40	594	593	593,417,285,176	(Al-Yousef et al., 2020),(Abdel-Hameed et al., 2013)	✓	✓	✓
264	●● Delphinidin-3-*p*-coumaroyl-glucose-drv.	**13.00**	727	728	728,611,449,278,162,146,116	(Flamini, 2013)	✓	✓	✓
265	●● Malvidin-3-*p*-coumaroyl-glucose-drv.	13.03	785	786	639,477,454,308,162,146	(Flamini, 2013)	×	✓	✓
266	●● Delphnidine-3-O-(6-O-acetyl)-5-O-diglucoside	13.15	669	670	345	(Flamini, 2013)	×	✓	✓
267	● Quercetin-pentose-rhamnose	**13.16**	580	581	302,265,150	(Mancini et al., 2018)	✓	×	✓
268	●● Naringenin-7-O-rutinoside	13.20	580	581	271,177,151	(Abu-Reidah et al., 2015)	✓	×	✓
269	● Rutin(quercetin-rutinoside)	13.35	610	609	609,301	(Moghadamtousi et al., 2015)	✓	✓	✓
270	●●Diosmetin-pentose-glucoside	13.61	594	593	593,300	(Hassan et al., 2019)	✓	✓	✓
271	●● Acetyl chrysophanol-O-glucose-xylose	13.62	590	591	591,297,253,133	(Zhao et al., 2013)	✓	×	✓
272	●● Apigenin-6-*C*-acetyl-rhamnoside-glucose	13.77	620	621	559,455,293	(Ozarowski et al., 2018)	×	✓	✓
273	● Kaempferol/ luteolin-O-rutinoside	13.75	594	593	593,431,285	(Moghadamtousi et al., 2015) ,(Mancini et al., 2018)	✓	✓	✓
274	●Quercetin-di-glucoside	13.59	626	627	627,303	(Mancini et al., 2018)	✓	✓	✓
275	●● Acyl quercetin-rhamnose-glucose	13.81	650	651	607,485,407,302,162	(Ben Said et al., 2017)	✓	✓	✓
276	● Kaempferol-O-robinobioside	14.12	594	593	593,285	(Moghadamtousi et al., 2015)	✓	✓	✓
277	●● Luteolin/ kaempferol-diglucoside	14.23	610	609	609,285	(Gu et al., 2012)	✓	✓	✓
278	●● Methyl-kempferol-pentose-hexose	14.43	594	593	593,300,285,228	(Gu et al., 2012)	✓	✓	✓
279	●●Naringenin-di-glucoside	14.72	596	597	596,505,272,324	(Li et al., 2016)	✓	✓	✓
280	●●Isorhamntein-pentose-hexose	14.95	610	609	609,315,294	(El-Hawary et al., 2020)	✓	✓	✓
281	● Quercetin-3-O-neohisperoside	15.05	610	609	609,301	(Moghadamtousi et al., 2015)	✓	✓	✓
282	● Quercetin-3-O-robinoside	15.11	610	609	609,301	(Moghadamtousi et al., 2015)	✓	✓	✓
283	●● Delphnidin-3-O-rutinoside	15.21	611	612	611,465,303	(Abdel-Hameed et al., 2013)	×	✓	✓
284	●● Eriodyctoyl-7-O-rutinoside	15.60	596	597	308,298,163	(El-Sayed et al., 2017)	✓	✓	✓
285	●● Orientein-7-O-deoxy hexose	15.63	594	593	594,579,449,300,286	(Ozarowski et al., 2018)	✓	✓	✓
286	● Homo-orientin	15.71	448	449	448,286,228	(George et al., 2015)	✓	×	✓
287	●Uercetin-O-rhmnoside (quercetrin)	16.13	448	449	447,302,146	(Souza et al., 2018)	✓	×	✓
288	●Dihydrokaempferol-hexoside	16.15	450	449	449,285,162	(Moghadamtousi et al., 2015)	✓	×	✓

289	● Kaempferol-O-hexose	16.43	448	449	448,286	(Mancini et al., 2018)	✓	×	✓
290	●● Trihydroxy-6-methoxyflavonone-7-O-glucoside	16.44	464	465	465,303	(Lee et al., 2018)	×	✓	✓
291	●●Phloretin-O-hexoside	16.60	436	437	437,275,162	(Mena et al., 2012)	✓	✓	✓
292	●● Myricetin-3-O-rhamnoside	16.61	464	465	319,317,300,146	(Abu-Reidah et al., 2015)	×	✓	✓
293	●●Apigenin-acetyl glucoside	16.63	474	473	473,270,203	(Abdelaziz et al., 2020)	×	✓	✓
294	●● Kaempferol / luteolin-rhamnose	16.76	432	431	431,287(100%)	(Jiménez-González et al., 2018), (Larrazábal-Fuentes et al., 2020)	✓	×	✓
295	●●Chryoseriol-O-glucoside	16.78	462	463	463,301	(El-Sayed et al., 2017)	✓	✓	✓
296	● Quercetin-pentoside	16.78	434	433	433,300	(Mancini et al., 2018)	✓	✓	✓
297	● Quercetin-glucose/galactose	16.85	464	465	302	(Moghadamtousi et al., 2015), (Mancini et al., 2018)	×	✓	✓
298	●Dihydrokaempferol-hexoside	16.89	450	449	449,287,270	(Moghadamtousi et al., 2015)	✓	×	✓
299	●●3,3`,7`-trimethyl-sulfate myricetin	17.13	440	441	441,318,123	(Simirgiotis et al., 2017)	✓	✓	✓
300	●● Tricetin-4`,-O-glucoside	17.37	464	465	303,229,149	(Yisimayili et al., 2019)	×	✓	✓
301	●Epi(catechin)	17.50	290	291	290,150,136,108	(Moghadamtousi et al., 2015) ,(Mancini et al., 2018)	✓	×	✓
302	●●Formonontein	17.51	268	269	269	(Tan et al., 2020)	✓	✓	✓
303	●●Tangretin-drv.	17.53	740	739	739,371	(Coria-Téllez et al., 2019)	×	×	✓
304	●●Visidulin III-drv.	17.60	740	739	739,345	(Wang et al., 2018)	×	×	✓
305	●●Taxifolin-methylether	17.61	318	319	319,257,130	(Taamalli et al., 2015)	✓	✓	✓
306	●●3`,4`,7- tri-Hydroxy-flavanone	17.77	273	272	226,185,158,111	(Rini Vijayan and Raghu, 2019)	✓	✓	✓
307	●● Dimethyl quercetin drv.	17.80	616	617	617,330	(Tan et al., 2020)	✓	✓	✓
308	●Glycitein	17.88	284	283	283,266	(George et al., 2015),88	✓	✓	✓
309	●● Pinocembrin	17.95	256	255	255	(Wang et al., 2008)	✓	×	✓
310	●●Pelargonidin-dimethyl drv.	18.02	301	302	302,300	(Ben Said et al., 2017)	✓	✓	✓
311	●●Liquiritigenin	18.06	256	255	255	(Wang et al., 2008)	✓	×	✓
312	●Tangeretin	18.57	372	373	373,315,300	(Adesanwo et al., 2020)	✓	×	✓
313	●Diadzein	18.67	254	255	255,237,211,165,145	George et al., 2015)	✓	×	✓
314	●Taxifolin	18.69	304	303	303,178	George et al., 2015)	✓	×	✓
315	●●Methoxytaxifolin	18.35	334	333	333,303,287(100)	(Bielecka et al., 2021)	✓	✓	✓
316	●●Rhamnetin /isorhamnetin	18.65	316	315	315,300,284,151	(Jiménez-González et al., 2018)	✓	✓	✓
317	●●Methoxytetrahydroxyisoflavone	18.90	316	315	315,272,151	(Uysal et al., 2021)	✓	✓	✓
318	● Kaempferol	18.93	286	287	259,229,151	(George et al., 2015), (Oboh et al., 2015)	✓	✓	✓

319	⁕ Luteolin	19.06	286	287	269,243,151	(George et al., 2015), (Oboh et al., 2015)	✓	✓	✓
320	⁕⁕Myricetin	19.12	318	319	319,257,162,102	(Abu-Reidah et al., 2015)	✓	✓	✓
321	⁕Genistein	19.15	270	271	271,253,225,215	(George et al., 2015)	✓	✓	✓
322	⁕⁕CamellianinA	19.17	620	621	433,313	(Abu-Reidah et al., 2015)	×	✓	✓
323	⁕⁕tri-Hydroxy-methoxy flavone	19.24	300	301	301,286	(Wang et al., 2008)	✓	✓	✓
324	⁕⁕tri-Hydroxy-tri-methoxy flavone	19.26	360	361	361,343,283,225	(Boukhalkhal et al., 2020)	✓	✓	✓
325	⁕⁕3,5,7-tri-Methoxy flavone	19.52	312	313	313,271,236	(Haq et al., 2020)	✓	✓	✓
326	⁕⁕Chrysoeriol	19.66	300	301	301,284,268	(Abu-Reidah et al., 2015)	✓	✓	✓
327	⁕⁕Methoxy kaempferol-methyl ether	21.74	330	331	331,285	(Falcão et al., 2013)	✓	✓	✓
328	⁕ Quercetin	23.03	270	271	271,253	(Wang et al., 2008)	✓	✓	✓
392	⁕⁕Apigenin	23.57	302	303	273,229,151	(George et al., 2015), (Oboh et al., 2015)	✓	✓	✓
330	⁕Hesperitin	23.71	302	303	285,267,231	(George et al., 2015), (Oboh et al., 2015)	✓	✓	✓

⁕ Compounds previously identified in *Annona muricata*

⁕⁕Compounds identified for the first time in *Annona muricata*&*Annona genus*

✓New compounds identified in *Annona muricata*

♠ **E: Ethanol extract of the whole fruit , W: Water extract of the edible part of the fruit , C: Aqueous extract of the leaves capsule**

♠ **The yellow highlight refers to the compounds identified only in the aqueous extract of the leaves capsule**

♠ **The red colored lines refers to the new compounds identified in *A. muricata* in the ethanolic extract of fruit , water extract of the edible part of the fruit & aqueous extract of the leaves capsule**

Table S4. Phytochemical compounds**(Alks)** detected and characterized *A. muricata* in the ethanolic extract of fruit , water extract of the edible part of the fruit & aqueous extract of the leaves capsule by using HPLC–ESI -MS/MS in positive and negative ionization modes

No	Cpd-name	Rt	Mwt	M ±	Ms/Ms Fragment	Ref.	E	W	C
331	●2,4,6-tribromoaniline	1.64	329	330	298,172,115	(Gavamukulya et al., 2019)	✓	✓	✓
332	●●Squamolone	4.41	128	129	129,112	(Avula et al., 2018)	×	×	✓
333	●●●Corydine	5.50	341	342	342,192	(Avula et al., 2018)	✓	×	✓
334	●Norcoclaurine	5.52	271	272	272,255,240,161	(Avula et al., 2018)	×	✓	✓
335	●Coclaurine	5.84	285	286	269,175,108	(Moghadamtousi et al., 2015) ,(Avula et al., 2018)	✓	×	✓
336	●Reticuline	5.90	329	330	330,191	(Moghadamtousi et al., 2015),(Avula et al., 2018)	✓	✓	✓
337	●Atherosperminine	6.15	309	310	310,295	(Moghadamtousi et al., 2015), (Avula et al., 2018)	×	✓	✓
338	●●Actinodaphnine	6.23	311	312	312,263,235	(Rinaldi et al., 2017)	×	✓	✓
339	●Norushinsunine	7.31	281	282	282,265	(Avula et al., 2018)	✓	✓	✓
340	●Anolobine glycoside	7.60	443	442	265,247,235,217,162	(Avula et al., 2018)	✓	✓	✓
341	●Annonamine	7.77	296	297	297	(Moghadamtousi et al., 2015)	✓	✓	✓
342	●Annomuricine	7.85	329	330	331	(Moghadamtousi et al., 2015)	✓	✓	✓
343	●●Isopiline	8.78	297	298	297,265	(Justino et al., 2021)	×	×	✓
344	● Vinblastine	10.29	811	810	810	(Rady et al., 2018)	×	×	✓
345	●●●Phytosphinguasine	16.40	317	318	317,266,260	(Calixto et al., 2017)	✓	✓	✓
346	●Methylcoclaurine	16.72	299	300	300,277	(Moghadamtousi et al., 2015),	✓	✓	✓
347	●*N*-acetyl tryptamine	17.66	202	203	203,175,161,146,135	(Moghadamtousi et al., 2015)	✓	×	✓
348	● Dimethyl coclaurine	17.71	313	314	314,300,143,107	(Moghadamtousi et al., 2015)	✓	✓	✓
349	● Stepharine	18.15	297	298	297,146	(Moghadamtousi et al., 2015),(Coria-Téllez et al., 2019)	×	×	✓
350	●Coreximine	18.29	342	341	297,282,265,237,222,191	(Avula et al., 2018)	✓	✓	✓
351	●●●Corytuberine	18.83	327	328	328,251	(Moghadamtousi et al., 2015),<br>(Avula et al., 2018)	✓	✓	✓
352	●Anomurine	18.84	327	328	328,297	(Avula et al., 2018)	✓	✓	✓
353	●Norcorydine	18.87	343	342	342,175	(Moghadamtousi et al., 2015),(Avula et al., 2018)	✓	×	✓
354	●●●*Trans*-feruloyl tyramine	18.94	327	328	328,192	(Moghadamtousi et al., 2015,(Avula et al., 2018)	✓	✓	✓
355	●●●*Trans*-caffeoyl tyramine	19.17	313	314	314,178	(Avula et al., 2018)	✓	✓	✓
356	● DNJ (deoxynojirmycin)<br>●DMJ(deoxymannojirmycin)<br>●DMDP (dideoxy-imino-D-	19.24	299	300	254,163	(Avula et al., 2018)	✓	✓	✓

	mannitol)								
357	●(4-chlorophenyl)-[4-(3-chlorophenyl)2-[(z)-3-(dimethylamino)prop-1-eyl]quinolin-6-yl]-3-methylimmidazol-4-yl) methanol	19.22	163	164	164	(Adesanwo et al., 2020)	✓	×	✓
358	●Annonaine	19.35	543	544	543	(Gavamukulya et al., 2019)	✓	×	✓
359	●Nuciferine	20.85	266	265	266,265,249,219	(Moghadamtousi et al., 2015),(Justino et al., 2021)	✓	×	✓
360	●Xylopine	21.61	295	296	297,246,234	(Moghadamtousi et al., 2015),(Justino et al., 2021)	×	✓	✓
361	●●Nornuciferine	22.02	295	296	296,281,246	(Moghadamtousi et al., 2015),	×	✓	✓
362	●Anolobine	24.26	281	282	282,236,212,174	(Avula et al., 2018)	✓	✓	✓
363	●●●Muricinine	24.66	281	282	282,265,235	(Avula et al., 2018)	✓	×	✓
364	●●●Pronuciferine	24.68	313	314	298,163	(Avula et al., 2018)	✓	✓	✓
365	●Isolaurelrine	25.37	311	312	312,266	(Avula et al., 2018)	✓	✓	✓
366	●●Nordextromethorphan	26.62	309	310	310,279	(Moghadamtousi et al., 2015), (Avula et al., 2018)	×	✓	✓
367	●●●Corydalmine	26.93	257	256	256	(Adesanwo et al., 2020)	✓	✓	✓
368	●●●Corytenchine	27.80	341	342	342,265	(Avula et al., 2018)	✓	×	✓
369	●●●Magnoflorinedrv.	28.77	341	342	192,165	(Avula et al., 2018)	✓	×	✓
370	●●●Dimethylcoclaurinedrv.	28.97	432	431	431,342(100%),89	(Avula et al., 2018)	✓	×	✓
371	●Coreximine	30.11	380	381	381,313	(Moghadamtousi et al., 2015)	✓	✓	✓

● Compounds previously identified in *Annona muricata*
●●Compounds identified for the first time in *Annona muricata*
●●●Compounds identified for the first time in *Annona* genus

♠ **E: Ethanol extract of the whole fruit , W: Water extract of the edible part of the fruit , C: Aqueous extract of the leaves capsule**

♠ **The yellow highlight refers to the compounds identified only in the aqueous extract of the leaves capsule**

Table S5. Phytochemical compounds (miscellaneous) detected and characterized *A. muricata* in the ethanolic extract of fruit , water extract of the edible part of the fruit & aqueous extract of the leaves capsule by using HPLC–ESI-MS/MS in positive and negative ionization modes

No	Cpd-name	Type	Rt	Mwt	M ±	Ms/Ms Fragment	Ref.	E	W	C
372	●●● Sucrose	Suger	0.80	342	341	179,161,119,113,131	(Friščić et al., 2016)	✓	✓	✓
373	●●●Glucourinoids	sugers	5.14	630	631	547,375,,483,146	(El Sayed et al., 2016)	✓	×	✓
374	●●● Tyrosine	AA	5.62	181	180	180,146,117	(Beelders et al., 2014)	✓	✓	✓
375	●●●Secoisolariresinol	Ph.pro	7.58	362	361	362,178	(Mena et al., 2012)	✓	✓	✓
376	●●7-*O*-methyl oleoresin-pentacetate	Ess.oil	8.62	617	618	618,582,516,393,147	(El Sayed et al., 2016)	✓	×	✓
377	●●●Coumarin glycoside	Coum	8.98	308	309	309,147,162	(Tan et al., 2020)	✓	×	✓
378	●Triglyceride	FA	9.15	176	177	176	(Ragasa et al., 2014)	✓	✓	✓
379	●●Esculin-O-glucoside	Coum	9.34	340	341	341,178,163	(Al-Yousef et al., 2020)	✓	✓	✓
380	●Mangostin	Xanth.	9.79	410	409	409,341,365,326	(Melot et al., 2009)	✓	×	✓
381	● Aloe emodin	Anthrq.	11.12	270	271	241,225,211	(George et al., 2015)	✓	✓	✓
382	●●1,1-Dimethyl allyl scopoletin	Coum	12.28	260	261	261,161	(Adesanwo et al., 2020)	✓	×	✓
383	●●●Resverateroldrv.	Coum.	12.36	422	421	421,227	(Tan et al., 2020)	✓	✓	✓
384	●Citroside	MG	13.99	386	386	385	(Moghadamtousi et al., 2015)	✓	✓	✓
385	●Annoionol B	MG	14.03	244	245	245	(Moghadamtousi et al., 2015)	✓	✓	✓
386	●Annoionol C	MG	14.25	244	245	245	(Moghadamtousi et al., 2015)	✓	✓	✓
387	●Rosioside	MG	14.67	386	385	385	(Moghadamtousi et al., 2015)	✓	✓	✓
388	●Loliolide	MG	16.43	196	197	196	(Moghadamtousi et al., 2015)	✓	✓	✓
389	●(+)-Epiloliolide	MG	16.81	196	197	196	(Moghadamtousi et al., 2015)	✓	✓	✓
390	●●phillygenin-O-hexose-O-pentose	Lignan	17.12	666	667	667,373,534	(Ozarowski et al., 2018)	✓	✓	✓
391	●●●7-demethyl suberosin	Coum	17.52	230	231	231,137	(Larrazábal-Fuentes et al., 2020)	✓	✓	✓
392	●(2)-3-hexenyl-B-D-glucoside	MG	17.54	262	263	263	(Moghadamtousi et al., 2015)	✓	✓	✓
393	●(1S,2S,4R) trans-2-hydroxy-1,8 cineol-B-D-glucoside	MG	17.83	332	333	332	Moghadamtousi et al., 2015)	✓	×	✓
394	●●●Urolithin-B- drv.	Coum	18.58	340	341	341,213	(Yisimayili et al., 2019)	✓	✓	✓
395	●●●Chromone drv.	Coum	18.70	364	365	325	(Simirgiotis et al., 2017)	✓	✓	✓
396	●●●Pimarane diterp	Diterp.	18.84	330	331	331,229,205,128	(Abdelaziz et al., 2020)	✓	✓	✓
397	●●●Trijuganone A	Diterp	19.00	294	293	293,221,177	(Zhu et al., 2007)	✓	✓	✓

398	●●●15,16-dihydro tanshinone	Diterp.	19.72	278	279	170,149	(Zhu et al., 2007)	✓	×	✓
399	●●●1,2-dihydro tanshinone	Diterp.	19.95	278	279	200,149	(Zhu et al., 2007)	✓	×	✓
400	●●●2,3,19,23-tetra-OH-urs-12-en-28-oic-acid-glucose	Triterp	20.17	666	667	667,503,162	(Hou et al., 2002)	×	×	✓
401	●●●2,3,19-tri-OH—urs-12-en-28-oic-acid-glucose	Triterp	22.71	650	651	650,503,162	(Hou et al., 2002)	✓	✓	✓
402	●●●3-oxo-α-ionyl β-d-glucoside	HC	23.73	370	371	357,303,185,163	(Jia et al., 2017)	×	✓	✓
403	● Stigmasterol	Sterol	25.75	412	413	413	(Ragasa et al., 2014)	✓	✓	✓
404	●β-sitosterol	Sterol	26.03	414	415	415	(Ragasa et al., 2014)	✓	✓	✓
405	●●2-Chloroethyl lineolate	FA	27.66	342	343	342	(Adesanwo et al., 2020)	✓	✓	✓
406	●●● Linoleic acid methylester	FA	27.73	294	293	293,204	(Ibrahim, 2012)	✓	✓	✓
407	●Octadecanoic acid	FA	27.87	284	283	283	(Adesanwo et al., 2020)	✓	✓	✓
408	●●Kaur-16-ene	Sisquit.	29.70	272	273	272	(Adesanwo et al., 2020)	✓	✓	✓
409	●1,3-Tridecanediol diacetate	FA	30.11	300	301	301	(Coria-Téllez et al., 2019) ,(Adesanwo et al., 2020)	✓	✓	✓
410	●●9,10 dehydroisolongifolene	Terpene	30.33	202	203	203 (M+1)	(Adesanwo et al., 2020)	✓	×	✓
411	●● Oleic acid	FA	30.24	282	281	281	(Gomes et al., 2019)	✓	✓	✓
412	●Palmetic acid	FA	30.38	256	255	255	(Gomes et al., 2019)	✓	×	✓
413	● 8-heptadecene	HC	30.92	238	239	239	(Adesanwo et al., 2020, Coria-Téllez et al., 2019)	✓	×	✓
414	●●Nonanal	HC	31.24	142	143	143	(Adesanwo et al., 2020)	✓	✓	✓

●Compounds previously identified in *Annona muricata*
●●Compounds identified for the first time in *Annona muricata*
●●●Compounds identified for the first time in *Annona* genus

Alks: alkaloids   Coum: coumarin,   Diterp: diterpenoid , FA: fatty acids, HC: hydrocarbon,   Mg : megastimane , Sesquit: sesquiterpene, Triterp: triterpene, Xanth: xanthene

♠ **E: Ethanol extract of the whole fruit , W: Water extract of the edible part of the fruit , C: Aqueous extract of the leaves capsule**

♠ **The yellow hi light refers to the compounds identified only in the aqueous extract of the leaves capsule**

Figure S1 Some compounds characterised from *A. muricata* aqueous leaves capsules extract extracts

Acetogenins

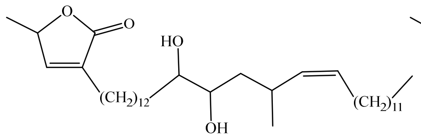

Cohibin A **(3)**

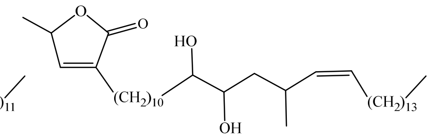

Cohibin B **(3)**

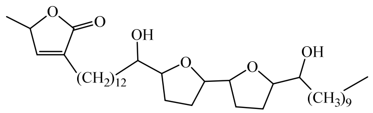

Deacetyl u varicin **(11)**

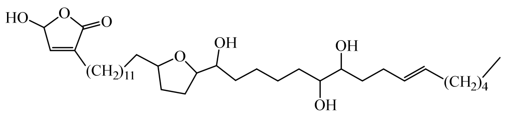

Muricin I **(14)**

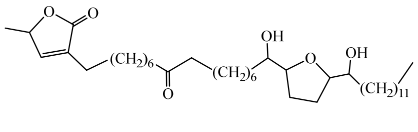

cis-reticulatacin-10-one **(15)**

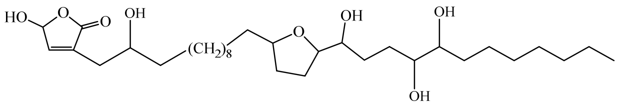

Muricin D **(51)**

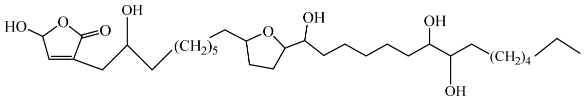

Muricin E **(60)**

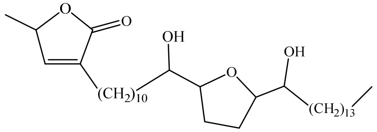

Panatellin **(126)**

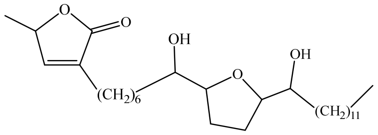

Solamin **(92)**

Phenolic compounds

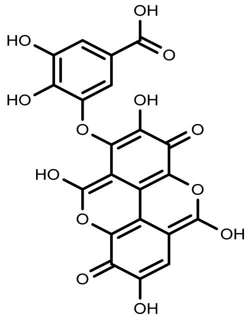

Sanguisorbic acid dilactone **(161)**

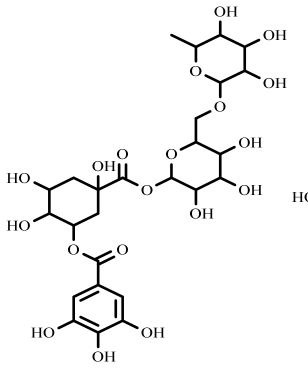

Galloyl-quinic acid -rutinoside**(164)**

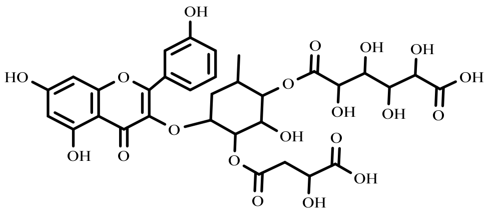

Mucic acid-Kaempferol-malic acid-rhamnose **(198)**

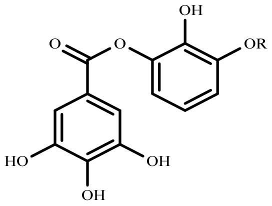

Galloyl-pyrogallol drv.**(209)**

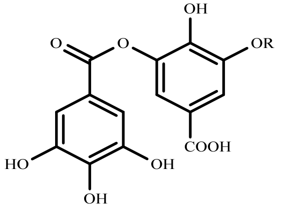

Digalic acid drv.**(220)**

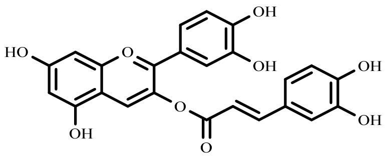

Catechin caffeic acid**(222)**

Flavonoids

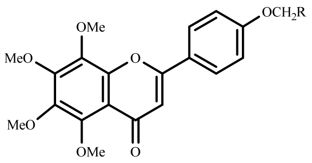

Tangretin-drv. **(303)**

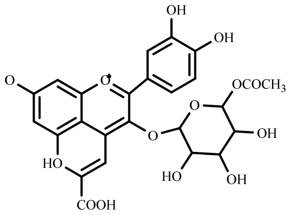

cyanidine-acetylglucoside pyruvic acid**(244)**

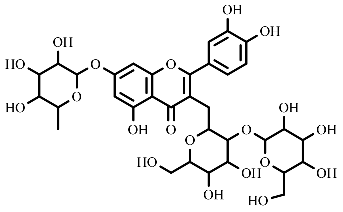

Quercetin-rhamnose-sophoroside **(258)**

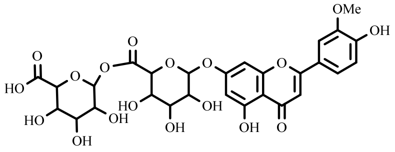

chrysoeriol-7-O-glucouronyl-glucouronic acid **(247)**

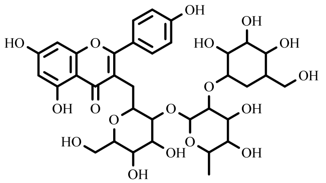

kaempferol-3-O-glucose-rhamnose-glucoside **(259)**

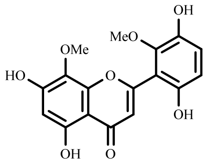

VisIdulin III-drv. **(304)**

Alkaoids

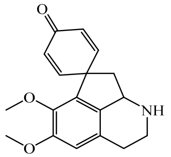

Stepharine **(349)**

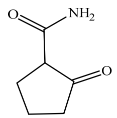

Squamolone **(332)**

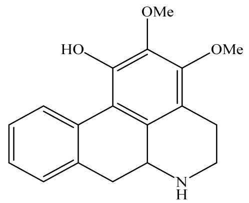

Isopiline **(343)**

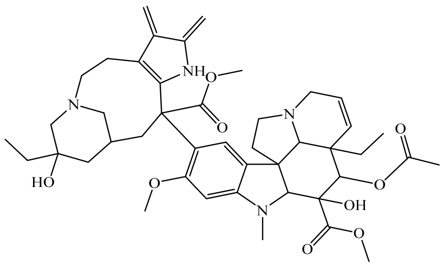

Vinblastine **(344)**

Lignan

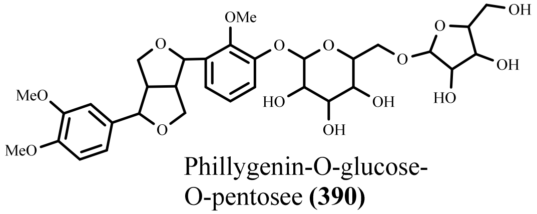

Phillygenin-O-glucose-
O-pentosee **(390)**

Triterpene

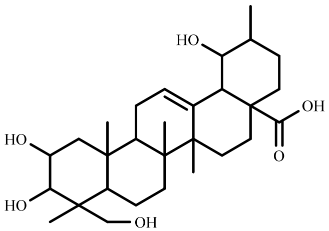

2,3,19,23-tetra-OH-urs-12en-
28oic-acid-glucose **(400)**

Figure S2: Photomicrograph of the recorded histopathological changes in the tumor mass and hepatic tissues of different experimental groups

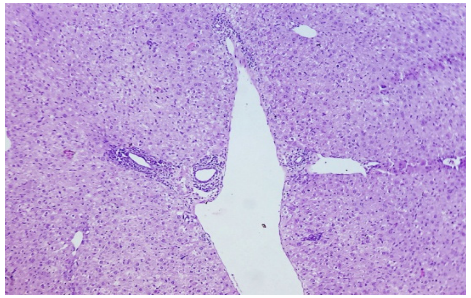

1-negative control liver cells

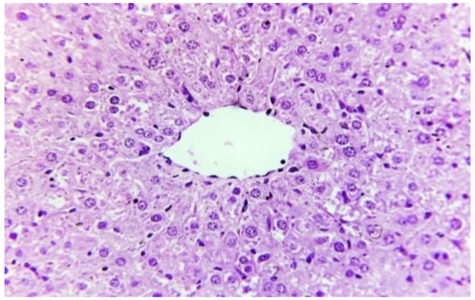

2- negative control liver cells

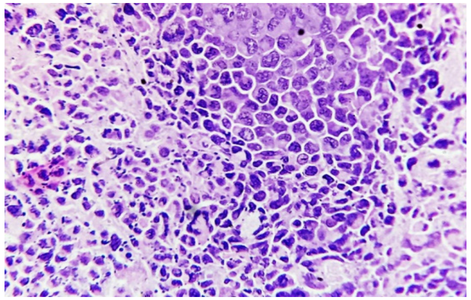

3-negative control tumor mass

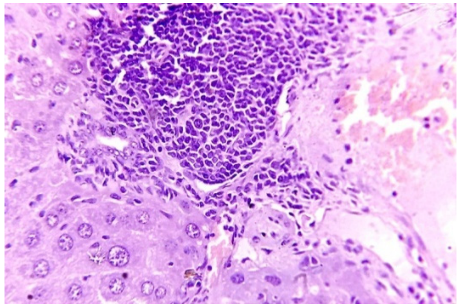

4-positive control liver cells

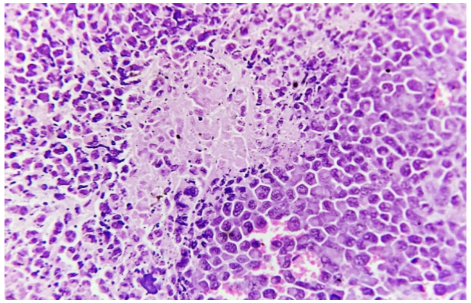

5- tumor mass-leaf extract treatment

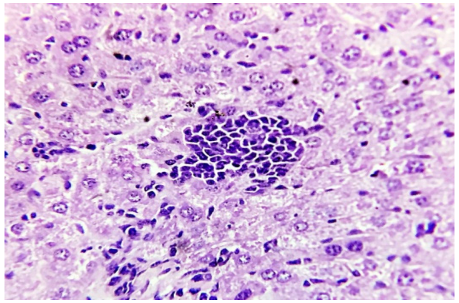

6- liver cells- leaf extract treatment

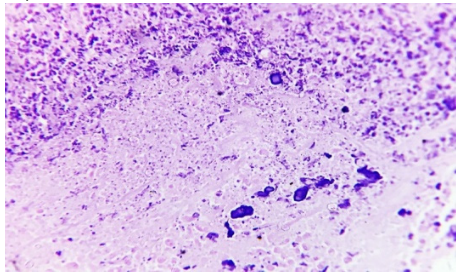

7-tumor mass-cisplatin treatment

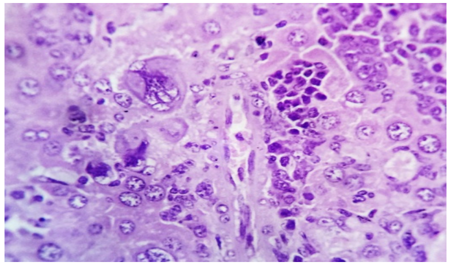

8- liver cells- cisplatin treatment

Figure S3: **Photomicrograph of the recorded immuonohistopathological changes in the tumor mass and hepatic tissues of different experimental groups**

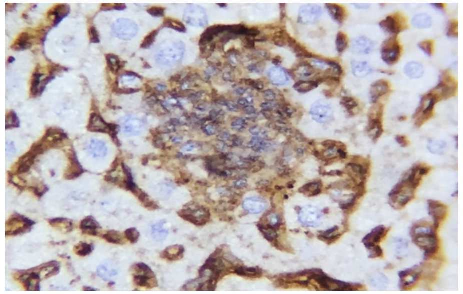

1-ck. tumor free liver

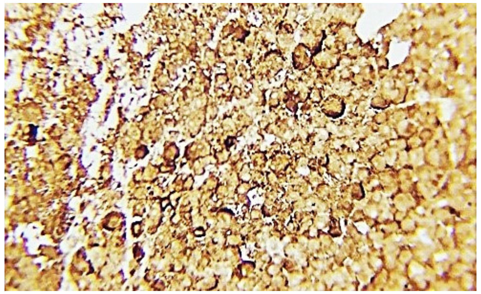

2- Cisplatin ck. tumor mass

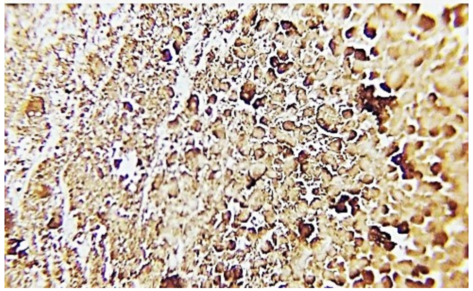

3- Cisplatin P53. tumor mass

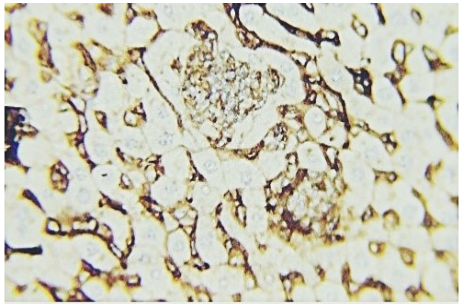

4-Cisplatin ck. liver metastasis

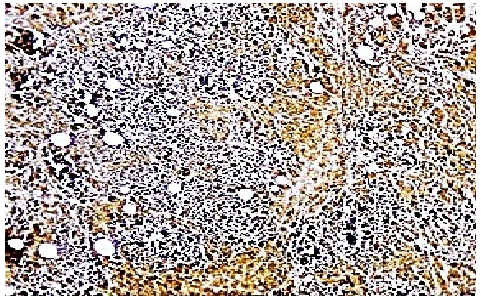

5-ck . control positive tumor mass

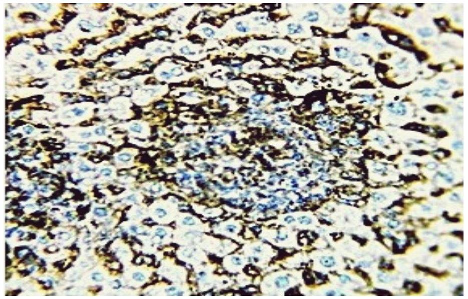

6-ck . control positive liver

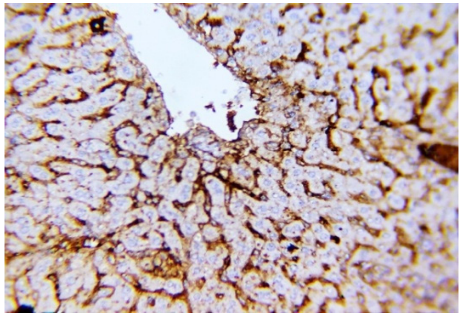

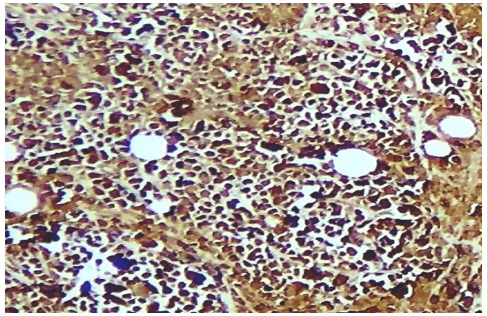

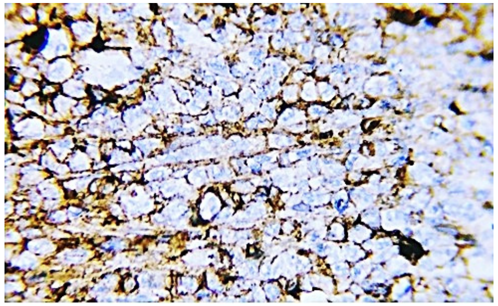

7-ck . control negative liver

8- ck . control positive tumor mass

9- P53 . control positive tumor mass

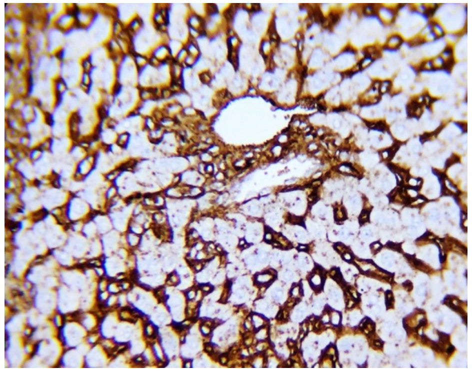

10-P53. control negative liver

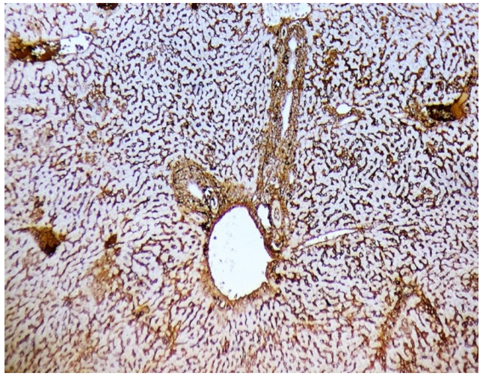

11- P53. control positive liver

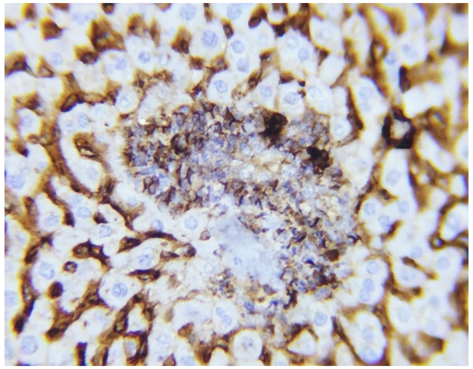

12- P53. liver metastasis

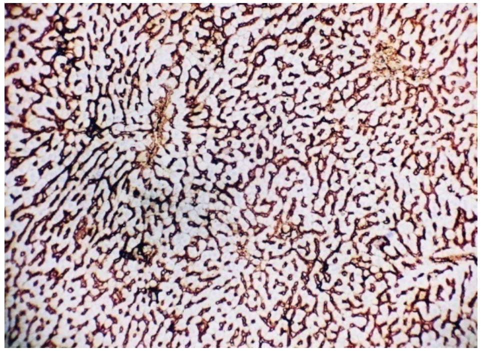

13-P53 Leaves extract treatment

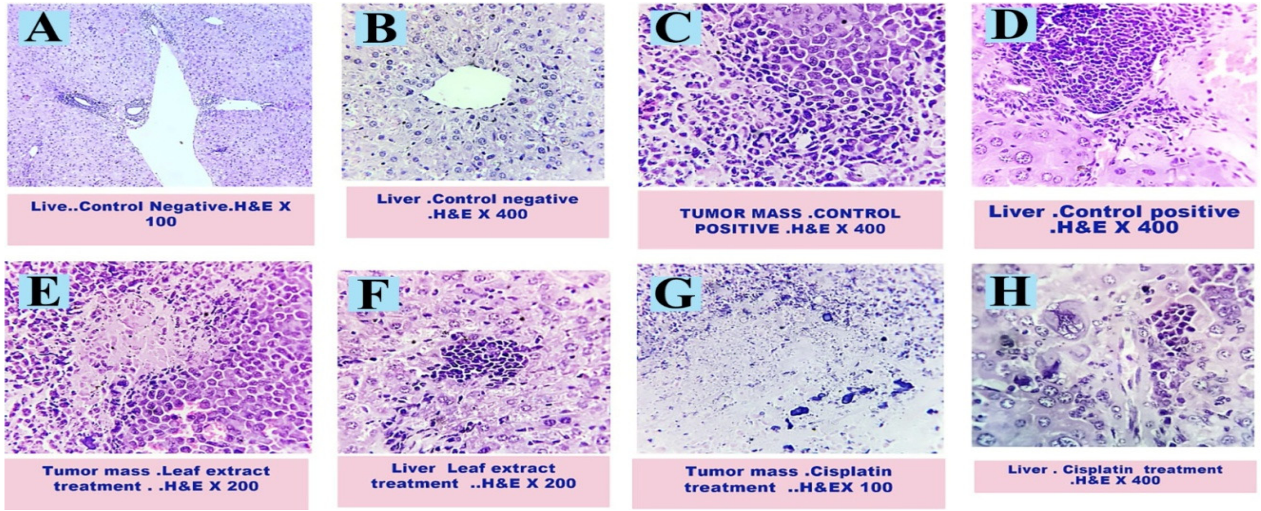
A
Live..Control Negative.H&E X 100
B
Liver .Control negative .H&E X 400
C
TUMOR MASS .CONTROL POSITIVE .H&E X 400
D
Liver .Control positive .H&E X 400
E
Tumor mass .Leaf extract treatment . .H&E X 200
F
Liver Leaf extract treatment ..H&E X 200
G
Tumor mass .Cisplatin treatment ..H&EX 100
H
Liver . Cisplatin treatment .H&E X 400

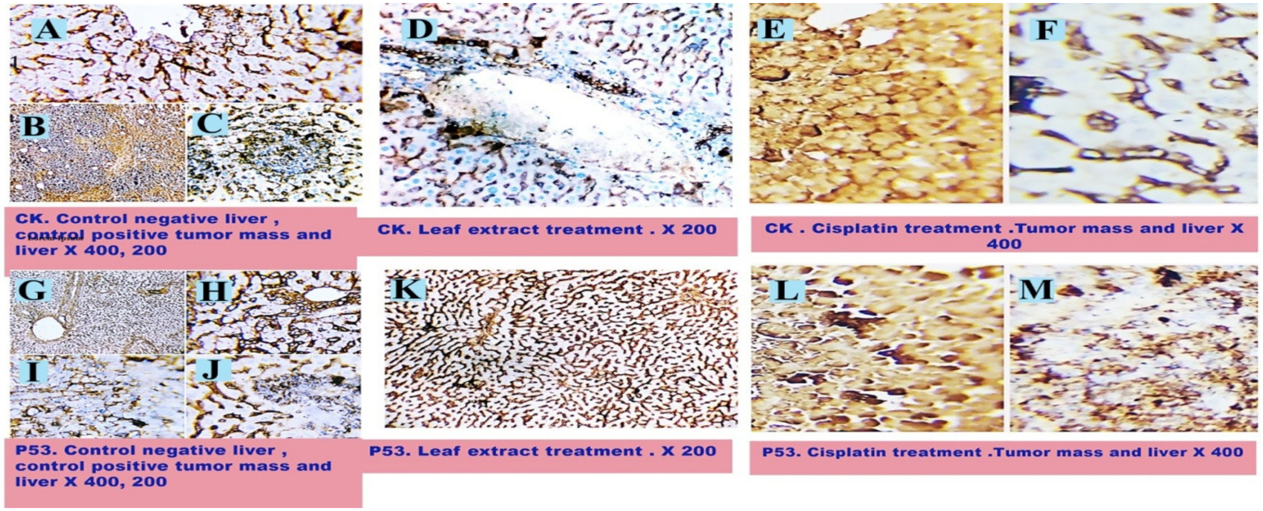

CK. Control negative liver , control positive tumor mass and liver X 400, 200

CK. Leaf extract treatment . X 200

CK . Cisplatin treatment .Tumor mass and liver X 400

P53. Control negative liver , control positive tumor mass and liver X 400, 200

P53. Leaf extract treatment . X 200

P53. Cisplatin treatment .Tumor mass and liver X 400
